# Effects of Tranexamic Acid on Hemorrhage Control and Deep Venous Thrombosis Rate After Total Knee Arthroplasty: A Systematic Review and Network Meta-Analysis of Randomized Controlled Trials

**DOI:** 10.3389/fphar.2021.639694

**Published:** 2021-07-21

**Authors:** Tao Ling, Zhihu Zhao, Wenwen Xu, Weihong Ge, Lingli Huang

**Affiliations:** ^1^ Department of Pharmacy, Suqian First Hospital, Suqian, China; ^2^ Department of Orthopaedics, Tianjin Hospital, Tianjin, China; ^3^ Department of Neurology, Nanjing Brain Hospital Affiliated to Nanjing Medical University, Nanjing, China; ^4^ Department of Pharmacy, Nanjing Drum Tower Hospital, The Affiliated Hospital of Nanjing University Medical School, Nanjing, China; ^5^ Department of Pharmacy, Jiangsu Cancer Hospital, Jiangsu Institute of Cancer Research, The Affiliated Cancer Hospital of Nanjing Medical University, Nanjing, China

**Keywords:** total knee arthroplasty, network meta-analysis, total blood loss, deep vein thrombosis, tranexamic acid

## Abstract

**Background:** Total knee arthroplasty (TKA) surgery has a lot of complications, especially hemorrhage, which can be controlled *via* tranexamic acid (TXA). The guidelines endorse the integration of TXA interventions in the management of TKA-induced complications. However, uncertainty surrounds the effects of different TXA therapies. This frequentist model network meta-analysis (NMA) aims to compare hemorrhage control and deep venous thrombosis (DVT) rate of different TXA therapies in TKA.

**Methods:** Articles were searched with the PubMed, Embase, Cochrane Library, and Web of Science from 1966 to October 2020. Randomized controlled trials (RCTs) comparing different TXA therapies, or with placebo in patients with TKA were included. Two investigators independently conducted article retrievals and data collection. The outcome was total blood loss and DVT rate. Effect size measures were mean differences (MDs), or odds ratios (ORs) with 95% confidence intervals (CIs). We conducted a random-effects NMA using a frequentist approach to estimate relative effects for all comparisons and rank treatments according to the mean rank and surface under the cumulative ranking curve values. All analyses were performed in Stata software or R software. The study protocol was registered with PROSPERO, number CRD42020202404.

**Results:** We identified 1 754 citations and included 81 studies with data for 9 987 patients with TKA. Overall, all TXA therapies were superior to placebo for total blood loss in TKA. Of all TXA therapies, M therapy (IV/IV infusion + oral TXA > 3g) was most effective for total blood loss (MD=−688.48, −1084.04–−328.93), followed by F therapy (IV TXA ≥ 15 mg/kg or 1 g three times). TXA therapies in this study are not associated with the increase of DVT risk.

**Conclusions:** TXA therapies in this study are effective and safe for the treatment of TKA-induced complications. M therapy (IV/IV infusion + oral TXA > 3 g) may be the most effective TXA therapy for hemorrhage control. TXA therapies in this study do not increase DVT risk. Considering hemorrhage control and DVT rate simultaneously, F therapy (IV TXA ≥ 15 mg/kg or 1 g three times) may be suggested to apply for TKA, and this study may provide a crucial clue to future TXA use.

## Introduction

Osteoarthritis (OA) is a major source of pain, disability, and socioeconomic costs worldwide and commonly affects athletes (Hunter and Bierma-Zeinstra, 2019). Total knee arthroplasty (TKA) is recommended for end-stage knee OA patients, but its safety concerns may outweigh the benefits (Juni et al., 2006). TKA surgery has many complications, especially hemorrhage, which can be controlled *via* the application of a pneumatic tourniquet, allogeneic transfusion, and antifibrinolytic therapy (Pawaskar et al., 2017; Arthur and Spangehl, 2019; Helito et al., 2019). Considering that tourniquet application during TKA is related to ischemic injury (Cao et al., 2018; Lei et al., 2019) and allogeneic blood transfusion has been associated with a poor postoperative outcome (Spahn, 2010), antifibrinolytic therapy may be a better choice.

Tranexamic acid (TXA) has attracted great interest in the past decade under the advantages, including ease of administration, low expense, and excellent hemostatic efficacy (Good et al., 2003). TXA is a synthetic lysin-analog that inhibits fibrinolysis by blocking the lysine-binding sites on plasminogen. Previous studies have identified that intravenous (IV) (Kuo et al., 2018; Zhang S. et al., 2019), intra-articular (IA) (Guzel et al., 2016; Pinsornsak et al., 2016), oral (Alipour et al., 2013; Cao et al., 2018), and combined TXA administration (Lin et al., 2015; King et al., 2019; Wang et al., 2019) can successfully reduce blood loss and transfusions in primary TKA without increasing the risk of thrombosis. The most common form of TXA administration during TKA is through IV administration, and IV administration is getting seriously due to reducing systemic exposure.

A recent network meta-analysis (Fillingham et al., 2018) showed that regardless of the formulation of TXA used, patients undergoing TKA showed a significant reduction in blood loss and risk of transfusion compared to placebo without clear difference between different formulations of TXA administration. However, this network meta-analysis did not involve specific intervention plans, and the study did not pay attention to TXA security issues. TKA is a risk factor for deep venous thrombosis (DVT), coupled with the anti-fibrinolysis effect of TXA, so it should be more cautious in clinical application. According to the guidelines for the prevention of DVT in Chinese orthopedic surgery, Doppler ultrasound has become the preferred imaging method for DVT as a noninvasive angiographic technique. Currently, numerous findings regarding TXA therapies have been generated, but no consensus has been reached on the optimal route and dosage of TXA administration. We undertook this systematic review and network meta-analysis of RCTs and ranked TXA therapies to compare the hemorrhage control and DVT rate of different TXA therapies in TKA.

## Materials and Methods

### Literature Search

Systematic literature searches were undertaken using PubMed, Embase, Cochrane Library, and Web of Science. Search strategies were used to identify relevant RCTs in patients with TKA from 1966 to October 2020. Without restrictions regarding year and language, the keywords combined with text terms followed by Boolean logical operators were conducted as an exhaustive search using “TXA,” “TA,” “tranexamic acid,” “total knee arthroplasty,” “knee arthroplasty,” “total knee replacement,” “knee replacement,” “TKA,” “TKR,” “randomized controlled trials” and “RCTs.” Furthermore, we scanned the bibliography lists of relevant previous studies aiming at conducting a recursive search for potential studies, and references of the retrieved papers and reviews were manually reviewed in case of the omission of relevant studies that were presented only with abstracts. This study was implemented and reported in accordance with the Preferred Reporting Items for Systematic Reviews and Meta-Analyses (PRISMA) extension statement for systematic reviews incorporating network meta-analyses for healthcare (Hutton et al., 2015). All analyses were based on previously published studies, and no ethical approval or patient consent was required.

### Inclusion/Exclusion Criteria

The inclusion criteria were as follows: 1) RCTs; 2) studies on patients with primary TKA; 3) studies comparing TXA therapies, or with placebo, thirteen TXA therapies were defined (A: IV TXA ≤ 10 mg/kg or 1 g once; B: IV TXA ≥ 15 mg/kg or 1 g once; C: IV TXA ≤ 10 mg/kg or 1 g twice; D: IV TXA ≥ 15 mg/kg or 1 g twice; E: IV TXA ≤ 10 mg/kg or 1 g three times; F: IV TXA ≥ 15 mg/kg or 1 g three times; G: IA TXA < 2 g; H: IA TXA ≥ 2 g; I: oral TXA ≤ 2 g; J: oral TXA > 2 g; K: IV/IV infusion + IA TXA ≤ 3 g; L: IV/IV infusion + IA TXA > 3 g; M: IV/IV infusion + oral TXA > 3 g); 4) studies reporting total blood loss or DVT rate outcomes in patients.

The following studies were excluded: 1) secondary analyses (review and meta-analysis), including some combined data analyses of published RCTs; 2) TXA combined with other drugs such as epinephrine, morphine, betadine, and so on; 3) abstract only (insufficient data); 4) not RCT; 5) case report.

### Data Extraction and Quality Assessment

Two reviewers independently extracted data from the included studies using a pre-designed excel data extraction form. We would pilot-test the form on a small number of articles. Disagreements would be resolved by consensus or a third reviewer. The Cochrane risk of the bias assessment tool was used to determine the methodological quality of RCTs. A total of six domains were evaluated: random sequence generation, allocation concealment, participant blinding, outcome assessor blinding, incomplete outcome data, and selective reporting. Each domain was assigned a judgment of low risk of bias, high risk of bias, or unclear risk of bias. The judgments for each domain were made strictly following the Cochrane Handbook V.5.1.0, Chapter 8.5 and Review Manager 5.3 software was used.

### Outcome Measures

The outcome was total blood loss and DVT rate. The total blood loss was calculated by the Gross and Nadler formula (Nadler et al., 1962; Ward et al., 1980), which was equal to the loss calculated from the change in Hematocrit plus the volume transfused when either reinfusion or allogeneic transfusion was performed. For total blood loss, the publication must have reported a standard deviation of 95% confidence interval at the last follow-up period. All of the patients in RCTs performed Doppler ultrasound by experienced ultrasound doctor to diagnose DVT before the operation and postoperatively. We extracted number of DVT among different interventions at the last follow-up period.

### Statistical Analysis

The NMA comparing total blood loss and the number of DVT among different interventions was performed on STATA 14.2 based upon the frequentist models of NMA and the network command (White, 2015; Shim et al., 2017). For continuous data, we estimated the mean difference (MD) with 95% confidence intervals (CIs). For categorical data, we estimated odds ratios (OR) with 95% CIs. Significant differences were identified when the 95% CI did not include 0 for MD or 1 for OR. The overall effect sizes (MDs or ORs) were generated from the median of the posterior distribution. In the analysis, “Placebo” was used as the reference group. Thirteen comparison groups were formed based on the available interventions. When trials contained three or more treatment arms, inconsistency was defined by the differences between direct and indirect effect estimates for the same comparison. The node-splitting approach and inconsistency model were used to test the consistency assumption (Dias et al., 2010). To rank the prognosis for all the groups, we used the surface under the cumulative ranking (SUCRA) values. Rankings for all evaluated treatments were based on the level of effect according to their posterior probabilities. SUCRA is equal to 100% for the best treatment and 0% for the worst treatment. Network meta-regressions were conducted to consider the potential impact of patients’ age, BMI, unilateral and the use of tourniquet (Salanti et al., 2009; Dias et al., 2013; Zeng et al., 2018). An inverted funnel plot was drawn for detecting the presence of publication bias. All statistical analyses were conducted using STATA software and R software.

## Results

### Study Selection and Characteristics of the Included Studies

**FIGURE 1:**
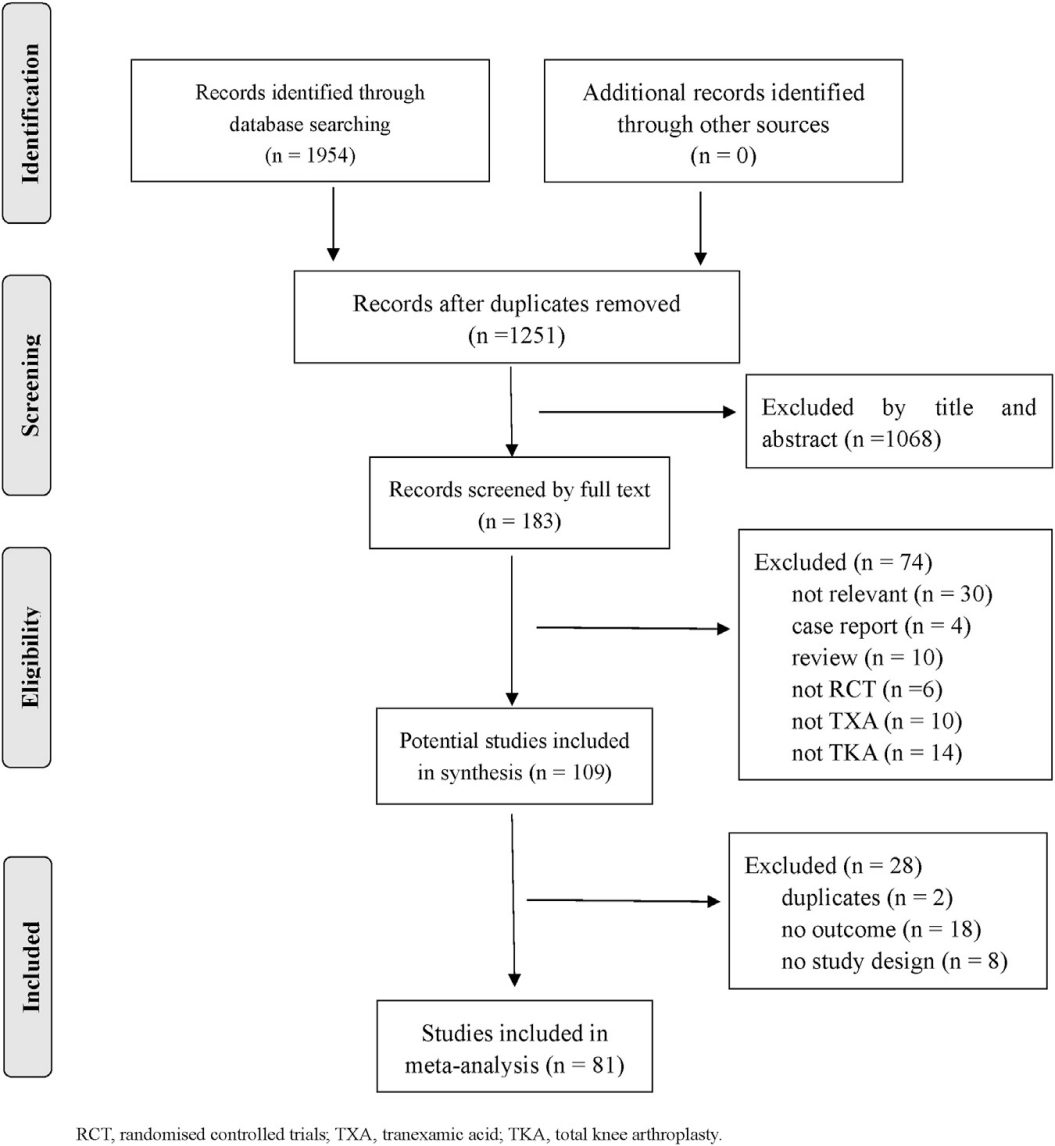
Literature review flow-chart.

Eighty one RCTs (9 987 patients) were included. Figure 1 depicts the details of the selection process. Thirteen TXA therapies were evaluated for patients with TKA in the RCTs. The characteristics of the comparisons and detailed information on the RCTs were shown in Supplementary Table 1. The methodological quality was evaluated for all included trials and was presented in Supplementary Figures 1, 2. The funnel plot indicated publication bias generation that depended on the asymmetrical distribution of scattering spots not symmetrical in the inverted funnel plot (shown in Supplementary Figures 3, 4).

### Effects on Total Blood Loss

**FIGURE 2:**
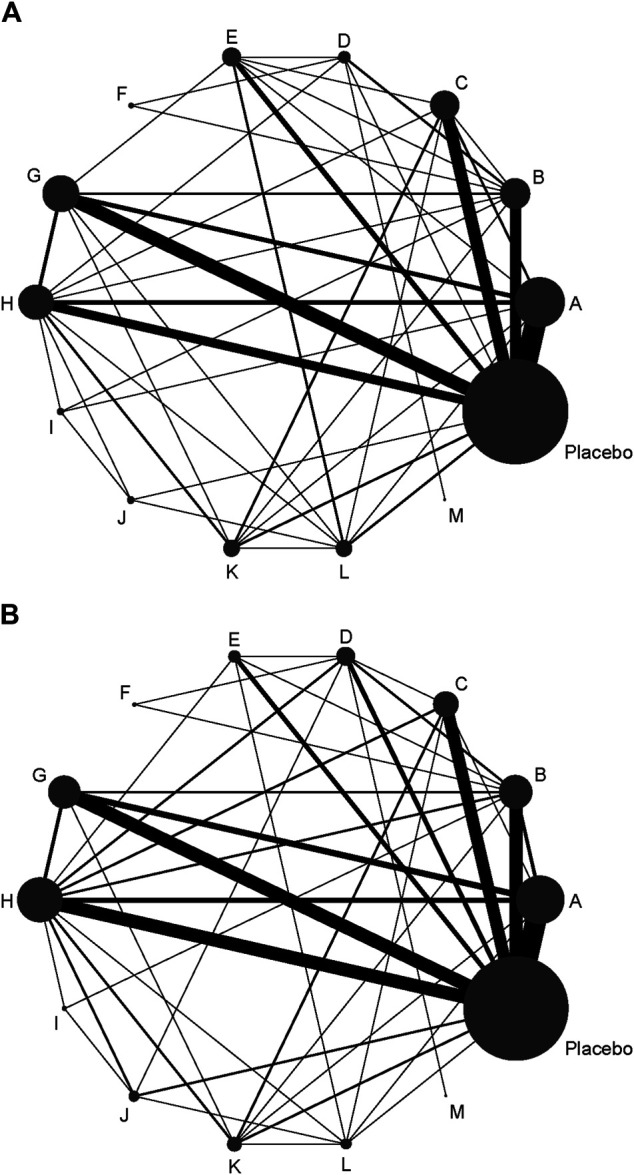
**(A)** The network of evidence of all the trials for total blood loss. **(B)** The network of evidence of all the trials for DVT rate.

**FIGURE 3:**
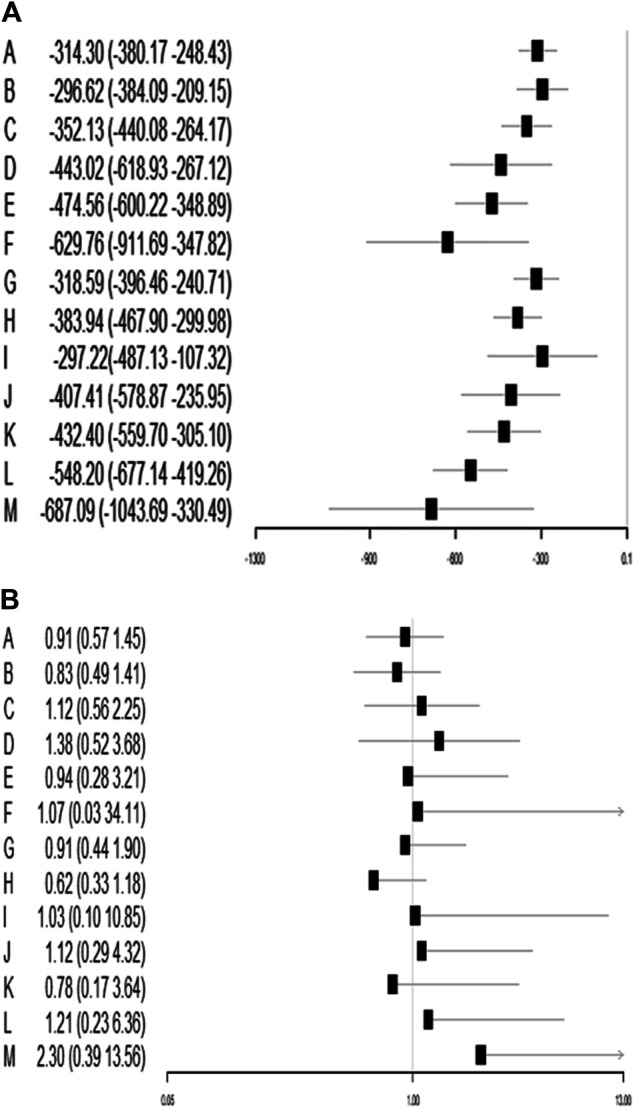
**(A)** Funnel plot of all the trials for total blood loss. **(B)** Funnel plot of all the trials for DVT rate.

A total of 72 trials (7 272 patients) involving all thirteen TXA therapies were analyzed. As shown in Figure 2A, the visual network geometry was conducted for displaying each arm. All results of the comparison were presented as the MD and 95% CIs. We compared total blood loss of all treatment regimens with that of placebo. A total of thirteen TXA therapies were statistically significant superior to placebo group and underlying estimates of effect presented with a relative wide (CIs), including M, F, L, E, D, K, J, H, C, A, G, I, B therapies (shown in Figure 3A).

Additionally, E and F therapies were significantly more effective than A therapy, B therapy, and G therapy. L therapy was significantly more effective than A therapy, B therapy, C therapy, H therapy, I therapy and G therapy. M therapy was significantly more effective than A therapy, B therapy, and G therapy. The detailed results were shown in Table 1. We conducted network meta-regressions, which showed that there might be significant interactions between total blood loss and age, while there were no significant interactions between total blood loss and BMI or the use of tourniquet or whether bilateral or unilateral TKA. (shown in Supplementary Table 2).

**TABLE 1:** Detailed results of network meta-analysis.

M	0.47 (0.01,19.90)	0.53 (0.05,5.71)	0.41 (0.05,3.17)	0.60 (0.14,2.63)	0.34 (0.03,3.47)	0.49 (0.06,3.95)	0.27 (0.04,1.70)	0.49 (0.08,3.13)	0.40 (0.06,2.69)	0.40 (0.06,2.45)	0.45 (0.03,8.03)	0.36 (0.06,2.22)	0.44 (0.07,2.57)
−57.33 (−471.71,357.04)	F	1.13 (0.02,51.34)	0.88 (0.02,33.14)	1.29 (0.04,40.43)	0.73 (0.02,31.53)	1.04 (0.03,40.83)	0.58 (0.02,19.16)	1.04 (0.03,35.01)	0.85 (0.02,29.02)	0.85 (0.03,27.53)	0.96 (0.02,60.71)	0.78 (0.02,24.47)	0.93 (0.03,29.63)
−138.89 (−514.34,236.56)	−81.56 (−387.88,224.76)	L	0.78 (0.11,5.29)	1.14 (0.18,7.34)	0.64 (0.08,5.53)	0.92 (0.13,6.61)	0.51 (0.09,2.88)	0.92 (0.16,5.34)	0.75 (0.12,4.54)	0.75 (0.14,4.07)	0.85 (0.05,14.11)	0.69 (0.12,3.82)	0.82 (0.16,4.32)
−212.53 (−581.41,156.34)	−155.20 (−455.99,145.59)	−73.64 (−228.51,81.23)	E	1.46 (0.36,6.00)	0.83 (0.12,5.77)	1.19 (0.20,6.92)	0.66 (0.17,2.53)	1.18 (0.30,4.75)	0.97 (0.23,3.99)	0.97 (0.26,3.52)	1.09 (0.08,15.04)	0.88 (0.24,3.20)	1.06 (0.31,3.60)
−244.07 (−554.28,66.14)	−186.73 (−461.47,88.00)	−105.17 (−316.69,106.35)	−31.53 (−231.15,168.09)	D	0.57 (0.09,3.40)	0.81 (0.18,3.57)	0.45 (0.15,1.34)	0.81 (0.26,2.52)	0.66 (0.20,2.22)	0.66 (0.23,1.92)	0.75 (0.06,8.90)	0.61 (0.21,1.72)	0.73 (0.27,1.93)
−254.69 (−629.57,120.19)	−197.36 (−501.51,106.80)	−115.80 (−283.67,52.08)	−42.16 (−214.42,130.11)	−10.62 (−221.13,199.88)	K	1.43 (0.19,10.71)	0.79 (0.16,3.96)	1.43 (0.29,7.16)	1.17 (0.22,6.13)	0.66 (0.23,1.92)	1.32 (0.08,21.32)	1.07 (0.22,5.15)	1.28 (0.27,5.95)
−279.68 (−672.15,112.79)	−222.34 (−548.88,104.19)	−140.78 (−333.00,51.43)	−67.14 (−272.89,138.60)	−35.61 (−276.06,204.83)	−24.99 (−231.98,182.00)	J	0.55 (0.14,2.26)	1.00 (0.22,4.44)	0.82 (0.18,3.74)	0.81 (0.20,3.34)	0.92 (0.09,9.67)	0.75 (0.18,3.07)	0.89 (0.23,3.45)
−303.15 (−663.89,57.59)	−245.81 (−534.73,43.10)	−164.26 (−307.23,−21.28) *	−90.62 (−235.67,54.44)	−59.08 (−243.24,125.07)	−48.46 (−187.33,90.41)	−23.47 (−198.82,151.88)	H	1.80 (0.74,4.38)	1.47 (0.59,3.65)	1.47 (0.70,3.09)	1.66 (0.15,18.01)	1.34 (0.62,2.93)	1.61 (0.85,3.05)
−334.96 (−700.45,30.52)	−277.63 (−570.64,15.39)	−196.07 (−343.26,−48.88) *	−122.43 (−268.42,23.56)	−90.90 (−284.18,102.39)	−80.27 (−219.35,58.81)	−55.28 (−244.41,133.84)	−31.81 (−146.69,83.06)	C	0.82 (0.30,2.22)	0.82 (0.36,1.85)	0.92 (0.08,10.60)	0.75 (0.32,1.76)	0.89 (0.45,1.80)
−368.50 (−730.93,−6.07) *	−311.17 (−600.29,−22.05) *	−229.61 (−371.86,−87.35) *	−155.97 (−296.74,−15.20) *	−124.44 (−311.88,63.00)	−113.81 (−253.63,26.00)	−88.82 (−272.22,94.57)	−65.35 (−166.39,35.68)	−33.54 (−146.97,79.89)	G	1.00 (0.44,2.26)	1.13 (0.10,13.10)	0.91 (0.38,2.17)	1.09 (0.53,2.28)
−372.79 (−733.76,−11.82) *	−315.46 (−603.13,−27.78) *	−233.90 (−370.75,−97.04) *	−160.26 (−296.10,−24.41) *	−128.72 (−313.32,55.87)	−118.10 (−253.87,17.67)	−93.11 (−271.00,84.78)	−69.64 (−164.01,24.73)	−37.83 (−141.32,65.67)	−4.29 (−94.90,86.33)	A	1.13 (0.10,12.27)	0.92 (0.49,1.70)	1.10 (0.69,1.75)
−389.87 (−789.45,9.72)	−332.53 (−666.58,1.52)	−250.97 (−470.64,−31.31) *	−177.33 (−399.98,45.32)	−145.80 (−397.69,106.09)	−135.18 (−357.23,86.88)	−110.19 (−322.95,102.58)	−86.72 (−280.28,106.85)	−54.90 (−260.76,150.95)	−21.36 (−221.20,178.47)	−17.08 (−208.12,173.97)	I	0.81 (0.08,8.51)	0.97 (0.09,10.18)
−390.47 (−746.39,−34.55) *	−333.13 (−607.75,−58.52) *	−251.57 (−401.73,−101.42) *	−177.93 (−321.87,−34.00) *	−146.40 (−320.92,28.11)	−135.78 (−278.37,6.81)	−110.79 (−298.03,76.45)	−87.32 (−199.77,25.13)	−55.51 (−173.73,62.72)	−21.97 (−129.99,86.06)	−17.68 (−122.82,87.46)	−0.60 (−198.99,197.78)	B	1.20 (0.71,2.02)
−687.09 (−1,043.69,−330.49) *	−629.76 (−911.69,−347.82) *	−548.20 (−677.14,−419.26) *	−474.56 (−600.22,−348.89) *	−443.02 (−618.93,−267.12) *	−432.40 (−559.70,−305.10) *	−407.41 (−578.87,−235.95) *	−383.94 (−467.90,−299.98) *	−352.13 (−440.08,−264.17) *	−318.59 (−396.46,−240.71) *	−314.30 (−380.17,−248.43) *	−297.22 (−487.13,−107.32) *	−296.62 (−384.09,−209.15) *	Placebo

A: IV TXA ≤ 10 mg/kg or 1 g once; B: IV TXA ≥ 15 mg/kg or 1 g once; C: IV TXA ≤ 10 mg/kg or 1 g twice; D: IV TXA ≥ 15 mg/kg or 1 g twice; E: IV TXA ≤ 10 mg/kg or 1 g three times; F: IV TXA ≥ 15 mg/kg or 1 g three times; G: IA TXA < 2 g; H: IA TXA ≥ 2 g; I: oral TXA ≤ 2 g; J: oral TXA > 2 g; K: IV/IV infusion+IA TXA ≤ 3 g; L: IV/IV infusion+IA TXA > 3 g; M: IV/IV infusion+oral TXA > 3 g.

A SUCRA line was drawn to rank the hierarchy of each TXA therapy (shown in Supplementary Figures 5, 6), M therapy had the largest probability of being the best treatment option (SUCRA = 91.3%), followed by F therapy (SUCRA = 88.5%). The rankings based on SUCRA were shown in Table 2.

**TABLE 2:** Rankings for all 13 TXA therapies based on SUCRAs.

Treatment	Total blood loss			DVT rate		
	MD (95%)	Sucra (%)	Rank	OR (95%)	Sucra (%)	Rank
Placebo	Reference	0.0	14	Reference	48.3	9
A	−314.30 (−380.17, −248.43)	25.5	11	0.91 (0.57,1.45)	55.4	4
B	−296.62 (−384.09, −209.15)	20.9	13	0.83 (0.49, 1.41)	62.1	2
C	−352.13 (−440.08, −264.17)	38.7	9	1.12 (0.56, 2.25)	42.5	12
D	−443.02 (−618.93, −267.12)	62.5	5	1.38 (0.52, 3.68)	33.5	13
E	−474.56 (−600.22, −348.89)	70.9	4	0.94 (0.28, 3.21)	53.6	6
F	−629.76 (−911.69, −347.82)	88.5	2	1.07 (0.03, 34.11)	49.2	8
G	−318.59 (−396.46, −240.71)	27.0	10	0.91 (0.44, 1.90)	55.2	5
H	−383.94 (−467.90, −299.98)	49.3	8	0.62 (0.33, 1.18)	79.2	1
I	−297.22 (−487.13, −107.32)	25.5	12	1.03 (0.10, 10.85)	50.2	7
J	−407.41 (−578.87, −235.95)	53.6	7	1.12 (0.29, 4.32)	46.2	10
K	−432.40 (−559.70, −305.10)	62.0	6	0.78 (0.17, 3.64)	60.5	3
L	−548.20 (−677.14, −419.26)	84.3	3	1.21 (0.23, 6.36)	43.3	11
M	−687.09 (−1,043.69, −330.49)	91.3	1	2.30 (0.39, 13.56)	20.7	14

A: IV TXA ≤ 10 mg/kg or 1 g once; B: IV TXA ≥ 15 mg/kg or 1 g once; C: IV TXA ≤ 10 mg/kg or 1 g twice; D: IV TXA ≥ 15 mg/kg or 1 g twice; E: IV TXA ≤ 10 mg/kg or 1 g three times; F: IV TXA ≥ 15 mg/kg or 1 g three times; G: IA TXA < 2 g; H: IA TXA ≥ 2 g; I: oral TXA ≤ 2 g; J: oral TXA > 2 g; K: IV/IV infusion+IA TXA ≤ 3 g; L: IV/IV infusion+IA TXA > 3 g; M: IV/IV infusion+oral TXA > 3 g.

### Effects on Deep Venous Thrombosis Rate

A total of 71 trials (8 501 patients) involving all thirteen TXA therapies were analyzed. As shown in the Figure 2B, the visual network geometry was conducted for displaying each arm. A comparison of results was presented by ORs and 95% CIs. We compared the safety of all treatment regimens with that of a placebo, but no significant difference was observed among them. The detailed results were shown in Table 1 and Figure 3B. H therapy had the largest probability of being the best treatment option (SUCRA = 79.20%). The rankings based on SUCRA were shown in Table 2.

### Inconsistency of Evidence

Results of the evaluation of the inconsistency for all comparisons and all details and original data of testing inconsistency were presented in Supplementary Figures 7, 8. We noted a significance level of *p* > 0.05 for all cases, which indicated that inconsistency was not present in any comparison. Due to the absence of statistically significant inconsistency between direct and indirect estimates explored by the node-splitting approach, it was applied for a valid comparison of the above-mentioned TXA interventions (shown in Supplementary Figures 9, 10).

## Discussion

**FIGURE 4:**
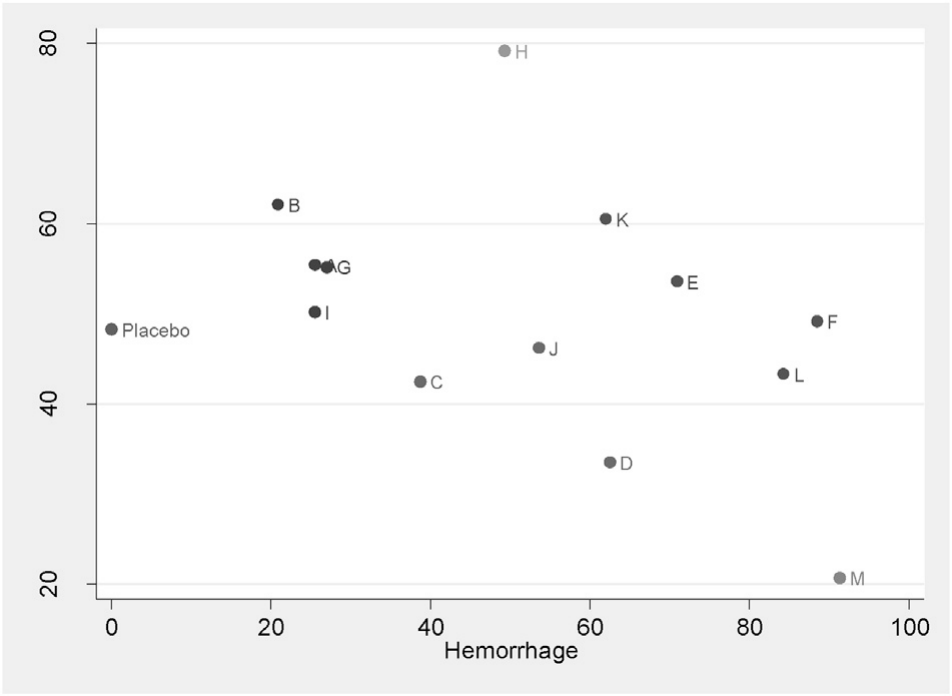
Clustergram of total blood loss and DVT risk.

This is the first network meta-analysis to take all available evidence into account from RCTs directly or indirectly comparing specific TXA therapies for TKA use, thereby increasing the power of the study. The main findings are: 1) TXA therapies are effective for the blood management after TKA; 2) The highest probability of being the best intervention for total blood loss in TKA is probably M therapy (IV/IV infusion + oral TXA > 3 g) (SUCRA = 91.3%); 3) E therapy (IV TXA ≤ 10 mg/kg or 1 g three times), F therapy (IV TXA ≥ 15 mg/kg or 1 g three times), L therapy (IV/IV infusion + IA TXA > 3 g), and M therapy (IV/IV infusion + oral TXA > 3 g) are significantly more effective than A therapy, B therapy (IV TXA ≤ 10 mg/kg or 1 g once and IV TXA ≥ 15 mg/kg or 1 g once) and G therapy (IA TXA < 2 g) for blood loss reduction; 4) L therapy (IV/IV infusion + IA TXA > 3 g) is significantly more effective than G therapy (IA TXA < 2 g), H therapy (IA TXA ≥ 2 g) therapy (IA TXA only) and I therapy (oral TXA ≤ 2 g) for total blood loss reduction; 5) All the TXA therapies in this study are independent with DVT risk; 6) Based on the clustergram of total blood loss and DVT risk (shown in Figure 4), F therapy (IV TXA ≥ 15 mg/kg or 1 g three times) is suggested for TKA according to the surface under SUCRAs considering the hemorrhage control and DVT rate simultaneously.

Our NMA of TXA therapies for patients with TKA demonstrated that all available therapies of TXA were significantly effective for the treatment of total blood loss. This evidence is reinforced by a previous network meta-analysis (Fillingham et al., 2018), as well as many conventional meta-analyses (Liu et al., 2017; Guo et al., 2018; Moskal and Capps, 2018; Meng et al., 2019). The highest probability of being the best intervention as for total blood loss control in TKA is probably M therapy (IV/IV infusion + oral TXA > 3 g), but little data regarding M therapy (IV/IV infusion + oral TXA > 3 g) exist. Only one double-blinded trial, M therapy (IV/IV infusion + oral TXA > 3 g) was IV TXA 20 mg/kg twice, and then oral 1 g TXA from postoperative day (POD) 1 to POD 14, which showed that IV and subsequent long-term oral TXA produced less blood loss compared with short-term TXA without increasing the risk of complications (Wang et al., 2019). The second highest probability of being the best intervention for total blood loss control in TKA is F therapy (IV TXA ≥ 15 mg/kg or 1 g three times). Only one trial in our study associated with F therapy (IV TXA ≥ 15 mg/kg or 1 g three times) in TKA was included, in which three doses of IV TXA can effectively and safely reduce TKA-induced total blood loss (Tzatzairis et al., 2019). In terms of dosage, total dose of two therapies is more than 3 g. And the higher the total dose of TXA, the better it performed. Therefore, M and F therapies are effective for hemorrhage control. In terms of delivery method, both M and F therapies include IV administration. And oral administration is also included in M therapy. IV or oral TXA is both absorbed through the systemic circulation, and exposure level is very high, which is the material basis for the efficacy.

E therapy (IV TXA ≤ 10 mg/kg or 1 g three times) is also three doses of IV TXA, and the single dose of TXA as for E therapy (IV TXA ≤ 10 mg/kg or 1 g three times) is less than or equal to 1 g or 10 mg/kg. Five trials (Maniar et al., 2012; Shinde et al., 2015; Song et al., 2017; Sun et al., 2017; Adravanti et al., 2018) in our study associated with E therapy (IV TXA ≤ 10 mg/kg or 1 g three times) in TKA from 2012 to 2018 were included. Both E therapy (IV TXA ≤ 10 mg/kg or 1 g three times) and F therapy (IV TXA ≥ 15 mg/kg or 1 g three times) were significantly more effective than A therapy (IV TXA ≤ 10 mg/kg or 1 g once), B therapy (IV TXA ≥ 15 mg/kg or 1 g once) and G therapy (IA TXA < 2 g) in reducing blood loss. A clinical trial (Sun et al., 2017) comparing E therapy (IV TXA ≤ 10 mg/kg or 1 g three times) and B therapy (IV TXA ≥ 15 mg/kg or 1 g once) proved that E therapy (IV TXA ≤ 10 mg/kg or 1 g three times) was superior to B therapy (IV TXA ≥ 15 mg/kg or 1 g once) in reducing blood loss in TKA. Based on the available literature, there is no statistical difference between E therapy (IV TXA ≤ 10 mg/kg or 1 g three times) and F therapy (IV TXA ≥ 15 mg/kg or 1 g three times) in terms of reducing blood loss, and we find that three doses of IV TXA may be better than a single dose of TXA for reducing blood loss reduction. However, this conclusion is inconsistent with the previous NMA results (Fillingham et al., 2018), in which higher doses and multiple doses of TXA are not necessary to reduce blood loss. Lei et al. (2018) found that multiple doses of IV TXA could further diminish hidden blood loss, and decrease maximum hemoglobin drop following total hip arthroplasty (THA), similar to our study result that multiple doses of IV TXA may necessary in TKA.

IV injection for patients undergoing TKA is the best method for rapidly raising and maintaining the therapeutic concentration of TXA in the knee and the time taken for maximum plasma levels of TXA to be reached has been reported to be 5–15 min for IV administration (Pilbrant et al., 1981; Svahn et al., 1986). Many studies have evaluated the timing of intravenous TXA administration in TKA, and several clinical studies have shown the efficacy of TXA when first or only given before surgery (Castro-Menéndez et al., 2016; Seviciu et al., 2016), during or before deflation of the tourniquet (Volquind et al., 2016; Prakash et al., 2017), or at the end of the surgery (Veien et al., 2002; Keyhani et al., 2016). It seems that it is better when TXA is first or only administered before operation (Tanaka et al., 2001; Fillingham et al., 2018).

Evidence including a growing number of well-conducted RCTs and summarized clinical guidelines have demonstrated that combined TXA plays a potentially important role in supporting the effectiveness of blood loss reduction in patients with TKA (Ou et al., 2018; Zhang Y. M. et al., 2019). L therapy (IV/IV infusion + IA TXA > 3 g) and M therapy (IV/IV infusion + oral TXA > 3 g) are both combined TXA and their total TXA dose is more than 3 g, in which the former is IV/IV infusion combined with IA TXA and the latter is IV/IV infusion combined with oral TXA. There was no statistical difference between L therapy (IV/IV infusion + IA TXA > 3 g) and M therapy (IV/IV infusion + oral TXA > 3 g) in terms of blood loss reduction. Thus, combined TXA (total dose 3>g) may be better than A and B therapies (IV TXA once), similar to E therapy (IV TXA ≤ 10 mg/kg or 1 g three times) and F therapy (IV TXA ≥ 15 mg/kg or 1 g three times). Only one trial in our study is associated with M therapy (IV/IV infusion + oral TXA > 3 g) in TKA, in which three doses of IV TXA could effectively and safely reduce blood loss undergoing TKA. We find that IV/IV infusion combined with IA TXA maybe have a better effect on blood loss reduction than IA TXA only. There is a trend toward better efficacy in reducing blood loss using combined TXA.

H therapy (IA TXA ≥ 2 g) had the largest probability of having the least DVT risk (SUCRA = 79.20%), which reminded that physicians may consider IA TXA in patients with a higher risk of DVT. This was supported by a recent NMA (Xu et al., 2019), which concluded that physicians may consider topical alone in patients with higher risk of thrombosis for its best safety profile. Continuous and high doses of administration of TXA could conceivably increase the rate of thromboembolic disease and IA isolated injection of TXA combined with drain clamping was reported first in the English literature (Ishida et al., 2011). The use of IA TXA injection has many theoretical and practical advantages. IA TXA may be a more specific method than a systemic route and using only a small amount of TXA in limited knee joint volume could create a high TXA concentration inside the knee joint. An animal study showed topical delivery of TXA had very low systemic absorption, which resulted in avoiding systemic side effects (Damji et al., 1998).

According to our study, low dose IA TXA does not associate with lower DVT risk, hence a high dose of TXA with no less than 2 g is suggested for TKA. These TXA therapies have shown their differential impacts based on their characteristics or their specific techniques. When it comes to IV TXA only, three doses of IV TXA and a single dose of 15 mg/kg TXA may be reasonable choices. When it occurs to combined TXA, IV/IV infusion combined with IA TXA and oral TXA are both fine and suggest their dose is more than 3 g.

Rather than only considering blood loss and DVT risk simultaneously, as the biggest strength, our NMA was pioneered to assess each specific TXA dosing schedule individually and compare all TXA therapies simultaneously for patients with TKA. Besides, the TXA therapies are complex and multifaceted and this is the first network meta-analysis associating with specific TXA therapies, which proves the particular significance of our NMA.

The limitations of our study also need to be acknowledged. Firstly, there was significant heterogeneity among studies. In a small number of articles included, drainage tube among the trials may have accounted for such heterogeneity. Secondly, the duration of the follow-up of the included studies was variable. Thirdly, none analysis for hemoglobin drop, drain blood loss, or hidden blood loss was conducted in this study. Fourthly, there was only one study involving M therapy (IV/IV infusion + oral TXA > 3 g), and the same for F therapy (IV TXA ≥ 15 mg/kg or 1 g three times). Based on the results of this study, more attention needs to be paid to M therapy (IV/IV infusion + oral TXA > 3 g) and F therapy (IV TXA ≥ 15 mg/kg or 1 g three times) in future studies.

## Conclusion

To summarize, our network meta-analysis indicates all thirteen TXA therapies are effective for hemorrhage control in TKA and no DVT risk is increased. The study provides some references that IV TXA ≥ 15 mg/kg or 1 g three times may be the optimal intervention for future studies.

## Data Availability

The original contributions presented in the study are included in the article/Supplementary Material, further inquiries can be directed to the corresponding author.
