# Supplementary material for: Effects of Tranexamic Acid on Hemorrhage Control and Deep Venous Thrombosis Rate After Total Knee Arthroplasty: A Systematic Review and Network Meta-Analysis of Randomized Controlled Trials
Source: Front Pharmacol. 2021 Jul 21;12:639694. doi: 10.3389/fphar.2021.639694 (PMC8335562; doi:10.3389/fphar.2021.639694)
Supplement: Supplementary file 2 [file Table1.docx]

Supplement Table 1. Characteristics of the included studies

| Study | Country | Group | Total dose | Sample | Time of administration | Unilateral | Tourniquet | Tourniquet time (min) | Gender (m/f) | Age | BMI (kg/m^2^) | Operation  (min) | Follow-up |
| --- | --- | --- | --- | --- | --- | --- | --- | --- | --- | --- | --- | --- | --- |
| Wang  2019 [1] | China | IV 20 mg/kg twice, and oral 1 g 14 times | < 18g | 60 | IV 20mg/kg 10 minutes before the surgery and 3 h after the operation, and then received oral drug from POD 1 to POD 14 | yes | no | **—** | 15/45 | 63.0±13.9 | 25.7±5.0 | 62.9±13.1 | 3m |
|  |  | IV 20 mg/kg twice | < 4g | 58 | before the surgery and 3 h after the operation |  |  |  | 11/47 | 64.1±9.3 | 25.5±3.9 | 63.6±12.4 |  |
| Tzatzairis2019 [2] | Greece | IV 15 mg/kg | < 1.5g | 60 | 10 min before incision, administered during the induction of the anesthesia | yes | no | **—** | 12/48 | 68.8±7.1 | 31.7±4.2 | 48.5±11.5 | 3m |
|  |  | IV 15 mg/kg twice | < 3g | 60 | 10 min before incision, and an additional dose 3 h after skin incision |  |  |  | 10/50 | 69.1±9.1 | 32.9±4.4 | 49.1±10.7 |  |
|  |  | IV 15 mg/kg three times | < 4.5g | 60 | 10 min before incision, and 3 h and 6 h after skin incision |  |  |  | 11/49 | 69.7±6.5 | 32.0±4.0 | 46.0±7.9 |  |
| Tang2019 [3] | China | oral 2 g | 2g | 50 | 2h preoperatively | yes | yes | NR | 7/43 | 65.5±7.4 | 25.5±3.4 | 73.0±10.7 | 0.5m |
|  |  | oral 2 g twice | 4 g | 50 | 2h preoperatively and 4h preoperatively |  |  |  | 12/38 | 63.3±7.7 | 25.2±3.9 | 72.3±8.4 |  |
|  |  | oral 2 g four times | 8 g | 51 | 2h preoperatively, at 4, 10, and 16h preoperatively |  |  |  | 9/42 | 66.2±7.8 | 25.8±3.9 | 73.2±13.5 |  |
| Tammachote2019 [4] | Thailand | IA 0.5 g | 0.5g | 40 | after fascial closure | yes | yes | 100±14.0 | 7/33 | 66.0±8.0 | 26.7±4.6 | NR | 4m |
|  |  | IA 3 g | 3g | 40 | after fascial closure |  |  | 100±16.0 | 7/33 | 67.0±10 | 27.0±4.2 |  |  |
| Nambiar2019 [5] | India | IV 10 mg/kg | < 1g | 44 | 20 min before the skin incision and just before tourniquet release | NR | yes | NR | 12/32 | NR | NR | NR | 3d |
|  |  | IV 15 mg/kg | < 1.5g | 44 | 20 min before the skin incision and just before tourniquet release |  |  |  | 18/26 |  |  |  |  |
| King2019 [6] | Australia | oral 1 g three times | 3g | 25 | 2 h prior to the commencement of surgery, 2h post-surgery and 6 h post-surgery | yes | no | **—** | 14/11 | 65.4±8.8 | 30.1±4.8 | 138.2±20.8 | 1.5m |
|  |  | topical 3 g+IV 1 g+oral 1 g | 5g | 28 | perioperatively, 2 h post-surgery and 6 h post-surgery |  |  |  | 12/16 | 63.8±9.7 | 30.2±4.6 | 140.5±21.4 |  |
| Wang2018 [7] | China | oral 2 g | 2g | 60 | 2 h before incision | yes | no | **—** | 18/42 | 63.9±13.1 | 25.3±4.17 | 66.6±11.0 | 3m |
|  |  | IV 20 mg/kg | < 2g | 60 | 5 min prior to incision |  |  |  | 15/45 | 66.9±9.48 | 25.0±3.41 | 67.6±12.5 |  |
|  |  | topical 2 g | 2g | 60 | following component implantation and before capsule closure |  |  |  | 16/44 | 63.2±11.8 | 25.5±3.8 | 67.8±12.9 |  |
| Wang2018 [8] | China | oral 2 g＋oral 1 g twice | 4g | 74 | 2 h before incision as a preoperative dose, 6 and 12 h after surgery | yes | no | **—** | 16/58 | 65.0±13.1 | 25.1±4.1 | 66.3±10.9 | 3m |
|  |  | topical 1.5 g twice | 3g | 73 | after components were cemented and the joint was thoroughly irrigated, and before capsule closure |  |  |  | 17/56 | 63.6±11.5 | 25.5±3.7 | 68.8±12.8 |  |
| Tsukada2018 [9] | Japan | IV 1 g twice＋IA 1 g | 3g | 75 | before skin incision and 6 h after the first dose, after implantation of the prosthesis and closing the capsule and retinaculum | yes | no | **—** | 18/57 | 77.0± 7.00 | 25.1 ± 3.2 | 92.0±12.0 | 7d |
|  |  | IV 1 g twice | 2g | 77 | before skin incision and 6 h after the first dose |  |  |  | 17/60 | 75.0 ± 8.00 | 25.2 ± 3.3 | 92.0±12.0 |  |
| Mehta2018 [10] | India | IV 1 g | 1g | 100 | before inflation of tourniquet | yes | yes | 86.26±8.83 | 41/59 | 62.9±6.08 | 26.5±5.58 | NR | 12d |
|  |  | IA 2.5 g | 2.5g | 100 | after wound closure through the drain pipe |  |  | 85±9.83 | 44/56 | 61.9±4.81 | 25.8±5.04 |  |  |
|  |  | placebo | 0g | 100 |  |  |  | 85.96±8.2 | 38/62 | 61.3±7.38 | 27.4±6.12 |  |  |
| Almeida2018 [11] | Brazil | IV 1 g | 1g | 51 | before the pneumatic cuff was inflated | NR | no | **—** | 15/36 | 66.6 | 25.2 | NR | 24h |
|  |  | placebo | 0g | 50 |  |  |  |  | 16/34 | 68.5 | 25.2 |  |  |
| Adravanti2018 [12] | Italy | IV 1 g three times | 3g | 50 | 30 min before induction of anesthesia and then at 3h and 9h after surgery | yes | yes | NR | NR | 70.9±9.6 | NR | NR |  |
|  |  | IV 1 g＋IA 3 g | 4g | 50 | 30 min before induction of anesthesia and then at 3h and 9h after surgery |  |  |  |  | 69.5±8.3 |  |  |  |
| Kwok2018 [13] | China | IA 1.5 g | 1.5g | 154 | after closure of arthrotomy | yes | yes | NR | 57/97 | 68.0±7.0 | 27.7 ± 4.00 | 93.0 ± 24.0 | 9d |
|  |  | placebo | 0g | 142 |  |  |  |  | 45/97 | 67.0 ± 7.00 | 28.3 ± 4.10 | 96.0 ± 24.0 |  |
| George2018 [14] | India | IV 10 mg/kg twice | ＜2g | 55 | at tourniquet release | yes | yes | 97.1 | 24/31 | 64.1 | 29.4 | 74.9 | 1.5m |
|  |  | IA 1.5 g | 1.5g | 58 | 10 min before the tourniquet inflation |  |  | 98.6 | 14/44 | 63.8 | 31.1 | 76.3 |  |
| Yuan2017 [15] | China | IV 20 mg/kg twice | ＜4g | 140 | 30 minutes before incising the skin, and the same dose 12 hours after TKA | yes | yes | 83.31±7.49 | 62/78 | 63.7±8.05 | 22.6±1.44 | NR | 3m |
|  |  | 3.0 g topical | 3g | 140 | administered after the subcutaneous tissue was sutured |  |  | 82.04±7.03 | 63/77 | 63.3±6.99 | 22.9±1.54 |  |  |
|  |  | oral 20 mg/kg twice | < 4g | 140 | 2 hours before the operation and the same dose 12 hours after TKA |  |  | 82.89±7.95 | 68/72 | 63.2±6.81 | 22.7±1.54 |  |  |
|  |  | placebo | 0g | 140 |  |  |  | 82.11±6.80 | 65/75 | 64.6±7.58 | 22.7±1.56 |  |  |
| Liu2018 [16] | China | IV 10 mg/kg | < 1g | 75 | before incision closure | yes | yes | NR | 34/41 | 63.6±6.55 | 26.6±3.42 | NR | 6m |
|  |  | IV 15 mg/kg | < 1.5g | 75 | before incision closure |  |  |  | 30/45 | 63.0±7.04 | 26.0±3.09 |  |  |
|  |  | placebo | 0g | 74 |  |  |  |  | 31/43 | 61.85±6.40 | 26.20±3.15 |  |  |
| Yen2017 [17] | China | IV 1g | 1g | 31 | 10 minutes before skin closure | yes | yes | NR | 4/27 | 69.13±7.94 | 28.1±4.53 | NR | 3m |
|  |  | IA 3g | 3g | 32 | aſter capsule closure before deflation of the tourniquet |  |  |  | 12/19 | 69.66±5.53 | 28.4±5.38 |  |  |
|  |  | placebo | 0g | 30 |  |  |  |  | 6/24 | 70.87±6.05 | 28.26±4.84 |  |  |
| Wang2017 [18] | China | IV 1 g | 1g | 100 | slow intravenous infusion 5 minutes before deflation of the tourniquet | yes | yes | NR | 26/74 | 68.19±6.62 | 28.16±4.19 | NR | 0.5m |
|  |  | placebo | 0g | 98 |  |  |  |  | 26/72 | 69.6±7.78 | 28.08±4.01 |  |  |
| Wang2017 [19] | China | IV 1g | 1g | 50 | administered right before skin closure | yes | yes | NR | 14/36 | 67.42±8.20 | 26.7±3.38 | NR | 1.25m |
|  |  | IA 1 g | 1g | 50 | before skin closure |  |  |  | 14/36 | 67.98±5.97 | 25.92±3.75 |  |  |
|  |  | placebo | 0g | 50 |  |  |  |  | 16/34 | 67.66±7.48 | 26.74±3.17 |  |  |
| Ugurlu2017 [20] | Turkey | IV 20 mg/kg | < 2g | 40 | administered IV 15 minutes before the tourniquet inflated | yes | yes | NR | 11/29 | 69.4±7.5 | 30.8±5.3 | NR | 2d |
|  |  | IA 3 g | 3g | 42 | administered with infiltration to the wound lips following suturing of the capsular incision |  |  |  | 9/33 | 70.6±8.6 | 31.1±5.4 |  |  |
|  |  | placebo | 0g | 41 |  |  |  |  | 6/35 | 66.4±6.6 | 30.2±3.8 |  |  |
| Sun2017 [21] | China | IV 30mg/kg | < 3g | 45 | infused 15 minutes before tourniquet inflation | yes | yes | NR | 13/32 | 67.2±8.1 | 25.8±5.6 | NR | 1m |
|  |  | IV 15mg/kg twice | < 3g | 45 | infused 15 minutes before tourniquet inflation and 3 hours postoperatively |  |  |  | 17/28 | 67.3±7.2 | 26.2±3.9 |  |  |
|  |  | IV 10mg/kg three times | < 3g | 45 | infused 15 minutes before tourniquet inflation, and 3 and 6 hours postoperatively |  |  |  | 10/35 | 68.1±7.9 | 26.1±4.9 |  |  |
|  |  | placebo | 0g | 45 |  |  |  |  | 8/37 | 67.4±8.4 | 25.9±5.2 |  |  |
| Stowers2017 [22] | New Zealand | IV 1.5 g | 1.5g | 51 | before release of tourniquet | yes | yes | NR | 27/24 | 71.0±8.6 | 31.2±5.5 | NR | 18m |
|  |  | IA 1.5 g | 1.5g | 60 | after implantation of prosthesis and closure of arthrotomy |  |  |  | 28/32 | 70.0±8.5 | 31.2±5.5 |  |  |
|  |  | placebo | 0g | 23 |  |  |  |  | 4/19 | 70.0±7.6 | 32.3±6.6 |  |  |
| Song2017 [23] | South Korea | IV 10mg/kg three times | < 3g | 50 | 20 min before tourniquet, 15 min before tourniquet deflation, 3 h after the second | yes | yes | 69±13.9 | 6/44 | 69.2±6.4 | 26.5±3.3 | NR | 3m |
|  |  | IA 1.5 g | 1.5 | 50 | after wound closure |  |  | 69.8±0.1 | 8/42 | 69.8±6.8 | 27.0±4.2 |  |  |
|  |  | IV 10 mg/kg three times＋IA 1.5 g | < 4.5g | 50 | 10 mg/kg similar to IV, IA after wound closure |  |  | 68.9±10.3 | 7/43 | 70.8±6.8 | 27.5±4.8 |  |  |
|  |  | placebo | 0g | 50 |  |  |  | 69.1±11.4 | 6/44 | 70.2±6.6 | 28.0±3.5 |  |  |
| Prakash2017 [24] | India, South Korea | IV 10 mg/kg three times | < 3g | 50 | 20 min before tourniquet, 15 min before deflation of tourniquet, 3 h after the second | yes | yes | NR | NR | 70.2 | NR | NR | 0.5m |
|  |  | topical 3 g | 3g | 50 | applied to joint cavity for 5 min prior to closure |  |  |  |  | 71.0 |  |  |  |
|  |  | IA 3 g | 3g | 50 | after wound closure |  |  |  |  | 68.6 |  |  |  |
|  |  | placebo | 0g | 50 |  |  |  |  |  | 68.3 |  |  |  |
| Lee2017 [25] | South Korea | IV 10 mg/kg twice | < 2g | 93 | 30 minutes before tourniquet deflation, the same dose repeated 3 h after surgery | yes | yes | NR | 6/87 | 73.4 ± 6.2 | 26.9 ± 3.6 | NR | 5d |
|  |  | IA 2 g | 2g | 93 | after closure of retinaculum and quadriceps tendon but before subcutaneous closure |  |  |  | 5/88 | 72.3 ±7.4 | 26.71 ±3.3 |  |  |
|  |  | IV 10 mg/kg twice＋IA 1 g | < 3g | 95 | 30 minutes before tourniquet deflation, the same dose repeated 3 h after surgery;  after closure of retinaculum and quadriceps tendon but before subcutaneous closure |  |  |  | 12/83 | 72.6 ± 6.3 | 26.7 ± 3.6 |  |  |
|  |  | IV 10 mg/kg twice＋IA 2 g | < 4g | 95 | 30 minutes before tourniquet deflation, the same dose repeated 3 h after surgery, and after closure of retinaculum and quadriceps tendon but before subcutaneous closure |  |  |  | 7/88 | 72.1 ± 6.9 | 27.7 ± 4.2 |  |  |
| Lee2017 [26] | China | oral 1 g three times | 3g | 94 | 2 h before induction of anesthesia、6 h and 12 h postoperatively | yes | yes | NR | 31/63 | 70.0±8.0 | 27.7±4.1 | 90±18 | 8.2m |
|  |  | placebo | 0g | 95 |  |  |  |  | 29/66 | 68.0±8.0 | 28.4±4.3 | 92±15 |  |
| Huang2017 [27] | China | IV 20 mg/kg+10 mg/kg four times + topical 1g | < 4g | 50 | 5 to 10 minutes before the skin incision and 3, 6, 12, and 24 hours later | yes | yes | NR | 18/32 | 66.2±8.3 | 25.1±1.5 | 51.8±4.6 | 6m |
|  |  | placebo (tourniquet only) | 0g | 50 |  |  |  |  | 15/35 | 65.8±6.8 | 24.7±1.3 | 50.7±5.2 |  |
| Xu2016 [28] | India | IA 2 g | 2g | 28 | at the end of the operation | yes | no | NR | NR | NR | NR | NR | 1m |
|  |  | IV 1 g | 1g | 40 | before the use of tourniquet |  |  |  |  |  |  |  |  |
|  |  | IA 2 g＋IV 1 g | 3g | 18 | before the use of tourniquet and at the end of the operation |  |  |  |  |  |  |  |  |
|  |  | placebo |  | 17 | did not undergo any treatment |  |  |  |  |  |  |  |  |
| Zekcer2016 [29] | Brazil | IV 20mg/kg | < 2g | 30 | given at the same time as anesthesia | yes | yes | 63 | 6/24 | 65.7 | NR | 77 | 0.5m |
|  |  | topical 1.5 g | 1.5g | 30 | sprayed over the area operated before tourniquet release |  |  | 63 | 9/21 | 65.7 |  |  |  |
|  |  | placebo | 0g | 30 |  |  |  | 63 | 5/25 | 65.7 |  |  |  |
| Volquind2016 [30] | Brasil | IV 2.5g | 2.5g | 32 | 5 minutes before deflation of the tourniquet | yes | yes | NR | 10/22 | 67.9±5 | NR | NR | 1m |
|  |  | placebo |  | 30 | 5 minutes before deflation of the tourniquet |  |  |  | 9/21 | 64.0±4 |  |  |  |
| Tzatzairis2016 [31] | Greece | IV 1 g | 1g | 40 | before incision (usually administered during the induction of the anesthesia) | yes | no | NR | 9/31 | 69.6±6.61 | 32.6±4.09 | 49.3±10.8 | 1.5m |
|  |  | IA 1 g | 1g | 40 | after joint capsule closure |  |  |  | 7/33 | 69.1±8.68 | 32.6±4.50 | 46.3±7.83 |  |
|  |  | placebo | 0g | 40 |  |  |  |  | 9/31 | 68.58±7.5 | 31.6±4.25 | 48.5±11.5 |  |
| Seviciu2016 [32] | USA | IV 20 mg/kg | < 2g | 29 | over 20 minutes starting at the beginning of surgery (skin incision) | yes | yes | NR | 13/14 | 65.7±8.6 | 32.3±7.2 | NR | 3d |
|  |  | placebo | 0g | 32 |  |  |  |  | 18/14 | 62.9±8.4 | 32.2±6.4 |  |  |
| Nielsen2016[33] | Denmark | IV 1g＋IA 3g | 4g | 30 | preoperatively and after closure of the capsule | yes | no | NR | 13/17 | 65.5±7.8 | 31.0 ± 4.2 | 52.7 ± 8.8 | 3m |
|  |  | IV 1g | 1g | 30 | preoperatively |  |  |  | 15/15 | 63.2 ± 8.6 | 28.2 ± 4.5 | 53.0 ± 11.7 |  |
| Keyhani2016 [34] | Iran | IV 0.5g | 0.5g | 40 | at the end of the surgery | yes | yes | NR | 26/14 | 68.4±10.4 | 32.7±5.5 | 87.0±12.0 | 2d |
|  |  | topical 1.5g + IA 1.5g | 3g | 40 | before joint closure, half used to irrigate the joint and the other half IA |  |  |  | 23/17 | 67±11.9 | 31.3±5.4 | 86.0±17.0 |  |
|  |  | placebo | 0g | 40 |  |  |  |  | 19/21 | 63.9±9.0 | 30.6±4.1 | 83.0±13.0 |  |
| Fillingham2016 [35] | USA | oral 1.95 g | 1.95g | 34 | 2 h prior to incision | yes | yes | NR | 13/21 | 62.0±11.0 | 33.0±7.0 | 82.0 ±14.0 | 5d |
|  |  | IV 1 g | 1g | 37 | prior to wound closure |  |  |  | 11/26 | 63.0±10.0 | 32.0±7.0 | 90.0 ±19.0 |  |
| Guzel2016 [36] | Turkey | IA 1.5 g | 1.5g | 50 | before closing the arthrotomy | yes | yes | NR | 7/43 | 66.5±5.1 | NR | NR | 10d |
|  |  | placebo | 0g | 50 |  |  |  |  | 10/40 | 67.0±4.5 |  |  |  |
| Drosos2016 [37] | Greece | IV 1 g | 1g | 30 | at the start of the wound suturing | yes | yes | 89.7±17.0 | 6/24 | 69.3±7.21 | 32.8±5.04 | NR | 1m |
|  |  | IA 1 g | 1g | 30 | at the start of the wound suturing |  |  | 90.6±15.1 | 6/24 | 71.1±6.32 | 33.4±6.08 |  |  |
|  |  | placebo | 0g | 30 |  |  |  | 94.8±15.6 | 6/24 | 71.8±6.5 | 32.6±4.37 |  |  |
| Chen2016 [38] | China | IV 1 g | 1g | 60 | 10 minutes before the tourniquet was inflated on the first knee for operation | no | yes | NR | 16/44 | 66.5±7.1 | 25.0±3.6 | 155.8±39.1 | 1.5m |
|  |  | placebo | 0g | 60 |  |  |  |  | 14/46 | 64.2±6.2 | 25.6±2.1 | 148.6±30.3 |  |
| Castro-Menendez2016 [39] | Spain | IV 1g TXA twice | 2g | 40 | intraoperatively and 3 h after surgery | yes | no | NR | 30/50 | 72.7±8.7 | 26.3±4.7 | NR | 2m |
|  |  | IV 2g TXA | 2g | 40 | 30 min before surgery |  |  |  | 30/50 |  |  |  |  |
|  |  | placebo | 0g | 80 |  |  |  |  | 29/51 |  |  |  |  |
| Yang2015 [40] | China | IV 0.5 g | 0.5g | 40 | applied into the joint for 5 min at the end of surgery | yes | yes | 101.3±11.6 | 12/28 | 69.0±5.0 | 25.0±4.0 | 95.5±14.2 | 0.5m |
|  |  | placebo | 0g | 40 |  |  |  | 96.3±12.7 | 10/30 | 67.0±6.0 | 24.0±4.0 | 93.0±13.9 |  |
| Wang2015 [41] | China | IV 1 g | 1g | 50 | injected after prosthesis implantation but before cavity close | yes | yes | NR | 25/25 | 52.6±12.4 | 26.2±2.17 | 58.2±10.6 | 0.25m |
|  |  | placebo | 0g | 50 |  |  |  |  | 22/28 | 53.2±10.2 | 25.6±3.03 | 60.8±9.87 |  |
| Wang2015 [42] | China | IV 0.5 g | 0.5g | 30 | immediately after skin closure | yes | yes | NR | 9/21 | 64.9±6.38 | 27.8±2.75 | NR | 2m |
|  |  | placebo | 0g | 30 |  |  |  |  | 6/24 | 65.0±6.75 | 28.3±3.28 |  |  |
| Shinde2015 [43] | Indian | IV 10 mg/kg three times | < 3g | 14 | the first dose inflation of the tourniquet after induction, the second dose was 4 h after the first dose, the third dose was after12 h of the first dose | yes | yes | 72.3±10.1 | 11/3 | 66.8±6.12 | 72.3±10.1 | NR | 10d |
|  |  | placebo | 0g | 14 |  |  |  | 73.6±10.4 | 12/2 | 63.8±8.85 | 73.6±10.4 |  |  |
|  |  | IV 10 mg/kg three times | < 3g | 14 | the first dose inflation of the tourniquet after induction, the second dose was 4 h after the first dose, the third dose was after12 h of the first dose | no | yes | 72.9±18.9 | 11/3 | 64.7±7.68 | 72.9±18.9 |  |  |
|  |  | placebo | 0g | 14 |  |  |  | 70.0±9.5 | 10/4 | 61.3±8.28 | 70.0±9.50 |  |  |
| Shen2015 [44] | China | IV 15 mg/kg | < 1.5g | 46 | at 15 min before the tourniquet was loosened | yes | yes | 91.4±21.2 | 8/33 | 65.7±8.2 | 25.4±5.2 | NR | 3m |
|  |  | placebo | 0g | 46 |  |  |  | 89.8±19.4 | 11/29 | 64.9±7.9 | 25.6±4.9 |  |  |
| Oztaş2015 [45] | Turkey | IV 15 mg/kg + 10mg/kg | < 2.5g | 30 | 15 mg/kg TXA was given 1 h before the inflation of the tourniquet and 1 h after the deflation of the tourniquet, IV 10 mg/kg TXA was given through one-hour infusion | yes | yes | NR | 5/25 | 68.6±5.38 | 30.6±4.52 | 115.9 ± 6.26 | 3m |
|  |  | IA 2g | 2g | 30 | in the final stage of the operation before the tourniquet deflation |  |  |  | 4/26 | 67.1±6.54 | 32.4±5.34 | 111.4 ± 7.66 |  |
|  |  | placebo | 0g | 30 |  |  |  |  | 5/25 | 67.0±6.15 | 32.4±4.55 | 116.2 ± 6.16 |  |
| Motififard2015 [46] | Iran | IV 0.5 g twice | 1g | 45 | once preoperatively and once 3 h postoperatively | yes | yes | NR | 10/35 | 67.04±8.01 | NR | NR | 6d |
|  |  | placebo | 0g | 45 |  |  |  |  | 13/32 | 65.66±4.97 |  |  |  |
| Lin2015 [47] | China | IA 1 g | 1g | 40 | after joint capsule closure | yes | yes | NR | 7/33 | 71.0±7.2 | 28.9±4.1 | 100.4±10.3 | 3m |
|  |  | IV 1g ＋IA 1 g | 2g | 40 | before skin incision and after joint capsule closure |  |  |  | 10/30 | 70.7±8.2 | 27.6±3.4 | 101.9±12.9 |  |
|  |  | placebo | 0g | 40 | after joint capsule closure |  |  |  | 5/35 | 69.7±8 | 24.5±3.5 | 105.8±13.2 |  |
| Kundu2015 [48] | India | IV 20 mg/kg | < 2g | 30 | after a test dose of 1 ml | yes | yes | 70.0±12.0 | 8/32 | 60.3±12.6 | NR | 159.3±22.9 | 10d |
|  |  | placebo | 0g | 30 |  |  |  | 72.0±10.0 | 7/23 | 59.6±12.1 |  | 152.0±14.8 |  |
| Karaaslan2015 [49] | Turkey | IV 15 mg/kg＋IA 3 g＋IV 30 mg/kg | < 7.5g | 41 | IV 15 mg/kg 10 minutes before the inflation of the tourniquet on the first side;  IA 3 g at 10 minutes before the deflation of the tourniquet.;  IV infusion of 10 mg/kg/h was continued for 3 h following completion on the second side | no | yes | NR | 8/32 | 65.6±7.0 | NR | 85.0±15.0 | 0.5m |
|  |  | placebo | 0g | 40 |  |  |  |  | 6/35 | 65.9±8.0 |  | 75.0±18.0 |  |
| Carvalho2015 [50] | Brazil | IA 1.5 g | 1.5g | 42 | applied into the knee before closure | yes | yes | NR | 18/24 | 70.8±6.5 | 29.5 | NR | 3m |
|  |  | IA 3 g | 3g | 40 | applied into the knee before closure |  |  |  | 7/31 | 70.0±8.2 | 30.2 |  |  |
|  |  | placebo | 0g | 43 |  |  |  |  | 10/30 | 69.3±6 | 29.9 |  |  |
| Aguilera2015 [51] | Spanish | IV 1 g twice | 2g | 50 | The first dose 15–30 min before tourniquet inflated, the second dose given 60–90 min after the first | yes | yes | 78.7±19.8 | 12/38 | 72.5±7.68 | 30.2±4.10 | 129.7±39.1 | 2m |
|  |  | topical 1 g | 1g | 50 | after prosthesis inserted and cemented, the entire operative field was rinsed and dried meticulously |  |  | 83.3±24.9 | 18/32 | 72.5±6.60 | 30.9±4.37 | 136.5±40.8 |  |
|  |  | placebo | 0g | 50 |  |  |  | 76.8±23.8 | 18/32 | 73.7±7.33 | 30.1±5.82 | 133.2±49.4 |  |
| Sarzaeem2014 [52] | Iran | IV 0.5g TXA | 0.5g | 50 | after closing the wound immediately. | yes | yes | NR | 7/43 | 66.9±7.2 | 31.6±2.70 | 94.0±11.2 | 2d |
|  |  | topical 3g TXA | 3g | 50 | the knee joint cavity irrigated with 3 g of TXA in 100cc of saline just before suturing for 5 minutes. |  |  |  | 7/43 | 68.1±6.8 | 32.3±3.40 | 99.5±12.4 |  |
|  |  | IA 1.5g TXA | 1.5g | 50 | immediately after wound closure，1.5 g of TXA in 100 cc of saline was injected through the drain |  |  |  | 6/44 | 67.5±7.6 | 30.7±3.50 | 97.4±13.6 |  |
|  |  | placebo | 0g | 50 |  |  |  |  | 7/43 | 66.8±8.2 | 32.6±2.90 | 110±13.1 |  |
| Patel2014 [53] | USA | IV 10 mg/kg TXA | < 1g | 42 | 10 minutes prior to tourniquet deflation. | yes | yes | 65.3±11.2 | 10/32 | 64.9±7.8 | 35.8±8.6 | NR | 6m |
|  |  | IA 2g TXA | 2g | 47 | directly into the surgical site and bathed in the solution for 2 minutes prior to tourniquet release |  |  | 68.6±9.70 | 13/34 | 64.8±9.7 | 32.7±7.0 |  |  |
| Levine2014 [54] | USA | IV 1g TXA | 1g | 20 | just prior to tourniquet release | NR | yes | NR | 7/13 | 62.3±7.31 | NR | NR | 6m |
|  |  | IV 20 mg/kg TXA | < 2g | 20 | just prior to tourniquet release |  |  |  | 8/12 | 66.5±9.65 |  |  |  |
|  |  | placebo | 0g | 25 |  |  |  |  | 10/15 | 62.4±10.1 |  |  |  |
| Kim2014 [55] | South Korea | IV 10 mg/kg twice | < 2g | 90 | 30 minutes before tourniquet deflation, and repeated 3h after the first injection | yes | yes | 86.7±17.0 | 11/79 | 73.5±5.5 | 26.3±3.4 | NR | 12m |
|  |  | placebo | 0g | 90 |  |  |  | 90.1±24.2 | 12/78 | 71.9±5.9 | 25.9±3.0 |  |  |
|  |  | IV 10 mg/kg twice | < 2g | 73 | 30 minutes before tourniquet deflation, and repeated 3h after the first injection | no | yes | 165.3±33.6 | 1/72 | 74.3±5.3 | 27.8±3.2 |  |  |
|  |  | placebo | 0g | 73 |  |  |  | 169.9±25.8 | 3/70 | 73.9±5.1 | 27.5±4.8 |  |  |
| Huang2014 [56] | China | IV 3g | 3g | 92 | before inflation of the tourniquet | yes | yes | NR | 37/55 | 65.4 ± 8.7 | 24.9 ± 3.2 | NR | 4d |
|  |  | IV 1.5g + IA 1.5 g | 3g | 92 | after implantation of the components and before inflation of the tourniquet. |  |  |  | 30/62 | 64.7 ± 9.5 | 25.2 ± 2.9 |  |  |
| Martin2014 [57] | USA | topical 2 g | 2g | 25 | after final washout, the wound was bathed in TXA for 2 minutes | yes | yes | NR | 14/11 | 67.2±10.6 | 35.1±7.92 | NR | 1m |
|  |  | placebo | 0g | 25 |  |  |  |  | 11/14 | 64.3±9.68 | 33.5±5.56 |  |  |
| Bidolegui2014 [58] | Argentina | IV 15mg/kg twice | < 3g | 25 | the first dose during induction of anesthesia and a second three hours later | yes | no | NR | NR | 71.5±9.4 | NR | 91.8±10.9 | 6m |
|  |  | placebo | 0g | 25 |  |  |  |  |  | 72±6.8 |  | 93.2±9.40 |  |
| Antinolf2014 [59] | Italy | IA 0.5 g | 0.5g | 20 | at the end of the surgical procedure | yes | yes | NR | 11/9 | 71.9±5.1 | NR | NR | 3m |
|  |  | placebo | 0g | 20 |  |  |  |  | 10/10 | 70.7±7.3 |  |  |  |
| Gomez-Barrena2014 [60] | Spain | IA 3g TXA | 3g | 39 | half administered by irrigation to achieve tissue impregnation before joint closure,  the other half IA after skin closure | yes | yes | 79.9±15.2 | 13/26 | 70.1±9.10 | 30.4±4.10 | 76.4±15.5 | 6m |
|  |  | IV 15 mg/kg TXA twice | < 3g | 39 | 15 to 20 minutes before tourniquet release and a second identical dose three hours after surgery |  |  | 80.5±14.4 | 14/27 | 71.8±10.3 | 30.2±4.20 | 75.1±14.1 |  |
| Seo2013 [61] | South Korea | IV 1.5 g | 1.5g | 50 | after closing surgical sites | yes | yes | NR | 6/44 | 66.8±6.3 | 28.1±3.1 | 54.1±1.40 | 2m |
|  |  | IA 1.5 g | 1.5g | 50 | while suturing |  |  |  | 5/45 | 67.5±6.6 | 27.8±3.5 | 54.6±14.3 |  |
|  |  | placebo | 0g | 50 |  |  |  |  | 5/45 | 67.8±6.1 | 27.9±3.3 | 55.1±1.40 |  |
| Sangasoongsong2013 [62] | Thailand | IA 0.5 g | 0.5g | 45 | after fascia closure | yes | yes | NR | 5/40 | 68.1±6.2 | 27.3±4.7 | 81.1±13.1 | 12m |
|  |  | IA 0.25 g | 0.25g | 45 | after fascia closure |  |  |  | 3/42 | 67.6±8.7 | 26.2±3.7 | 78.8±13.0 |  |
|  |  | placebo | 0g | 45 |  |  |  |  | 2/43 | 66.2±7.3 | 26.3±3.7 | 79.1±14.3 |  |
| Georgiadis2013 [63] | USA | topical 2.0 g | 2g | 50 | topically applied to the wound for 5 min after component placement and during cement hardening | yes | yes | 79.8±15.5 | 12/39 | 67.0±9.0 | 33.9±8.8 | NR | 1.5m |
|  |  | placebo | 0g | 51 |  |  |  | 81.4±14.0 | 19/31 | 64.5±8.2 | 34.2±5.9 |  |  |
| Lee2013 [64] | South Korea | IV 10mg/kg twice | < 2g | 36 | the first infusion after implantation before tourniquet release and the second infusion 6h after the first | yes | yes | 78.0 | 5/31 | 69.7±7.9 | 26.7 | NR | 3m |
|  |  | placebo | 0g | 36 |  |  |  | 78.0 | 5/31 | 69.2±7.7 | 26.9 |  |  |
| Alshryda2013 [65] | UK | topical 1 g | 1g | 79 | sprayed into the wound at the end of the total knee replacement | yes | yes | 74.0±10.2 | 30/79 | 65.5±9.60 | 32.2±5.93 | 74.0±10.2 | 3m |
|  |  | placebo | 0g | 78 |  |  |  | 72.0±10.2 | 44/78 | 67.1±10.2 | 31.1±5.03 | 72.0±10.2 |  |
| Aguilera2013 [66] | Spain | IV 1 g twice | 2g | 44 | 15 to 30 m before tourniquet inflated, and the second 60 to 90 minutes after the first dose | yes | yes | 85.7±22.0 | 4/40 | 72.4±6.6 | 30.9±3.9 | 85.4±23.0 | 2m |
|  |  | placebo | 0g | 43 |  |  |  | 95.71±19.0 | 7/36 | 74.9±7.0 | 29.8±8.5 | 95.5±19.2 |  |
| McConnell2012 [67] | UK | IV 10 mg/kg | < 1g | 22 | at induction of anaesthesia | NR | yes | NR | 10/12 | NR | NR | NR | 1m |
|  |  | placebo | 0g | 22 |  |  |  |  | 15/7 |  |  |  |  |
| Maniar2012 [68] | India | IV 10 mg/kg | < 1g | 41 | 15 minutes before deflation of the tourniquet | yes | yes | 79.0±10.7 | 10/30 | 67.3±9.1 | 29.4±5.5 | NR | 3m |
|  |  | IV 10 mg/kg twice | < 2g | 41 | 15 minutes before deflation of the tourniquet and 3 h after the first dose |  |  | 79.0±10.6 | 11/29 | 68.3±8.0 | 28.6±4.2 |  |  |
|  |  | IV 10 mg/kg twice | < 2g | 42 | 20 minutes before tourniquet inflation and 15 minutes before deflation of the tourniquet |  |  | 78.0±8.5 | 8/32 | 67.4±8.4 | 27.8±4.8 |  |  |
|  |  | IV 10 mg/kg three times | < 3g | 41 | 20 minutes before tourniquet application, 15 minutes before deflation of the tourniquet, and 3 h after the second dose |  |  | 80.0±14.3 | 7/33 | 66.8±7 | 31.2±7.3 |  |  |
|  |  | topical 3 g | 3g | 41 | after cementing the implant and before tourniquet release, at least 5 minutes of contact time was allowed before the tourniquet was deflated |  |  | 79.0±8.7 | 6/15 | 67.4±7.9 | 30.9±5.2 |  |  |
|  |  | placebo | 0g | 40 |  |  |  | 78.0±12.5 | 4/36 | 66.2±7.2 | 30.8±5.6 |  |  |
| Lin2012 [69] | China | IV 10 mg/kg | < 1g | 52 | 5 minutes before the skin incision and 5 minutes before deflation of the tourniquet | yes | yes | NR | 9/43 | 70.6±8.00 | 28.0±3.84 | NR | 7.5m |
|  |  | IV 10 mg/kg twice | < 2g | 49 | 5 minutes before the skin incision and 5 minutes before deflation of the tourniquet |  |  |  | 8/41 | 69.8±7.59 | 28.1±3.92 |  |  |
|  |  | placebo | 0g | 50 |  |  |  |  | 7/43 | 69.7±7.80 | 27.4±3.71 |  |  |
| Sangasoongsong2011 [70] | Thailand | IA 0.25 g | 0.25g | 24 | after fascia closure | yes | yes | NR | 2/22 | 69±8.2 | 27.0±3.4 | 115.5±16.2 | 6m |
|  |  | placebo | 0g | 24 |  |  |  |  | 6/18 | 69.2±7.6 | 26.8±4.1 | 123.7±23.3 |  |
| MacGillivray2011 [71] | United Arab Emirates | 10 mg/kg twice | < 2g | 20 | before deflation of the first tourniquet and 3 h after the first | no | yes | NR | 7/13 | 62.0±4.3 | 32.9 | NR | 4d |
|  |  | 15 mg/kg twice | < 3g | 20 | before deflation of the first tourniquet and 3 h after the first |  |  |  | 8/12 | 65.0±4.3 | 30.0 |  |  |
|  |  | placebo | 0g | 20 |  |  |  |  | 5/15 | 66.0±7.3 | 31.2 |  |  |
| Wong2010 [72] | Canada | IA 1.5 g | 1.5g | 31 | applied to the open joint surfaces after all components were cemented into place | yes | yes | 78.5±14.9 | 6/25 | 67±11.9 | 31.3±5.4 | 76.0±14.6 | 1.5m |
|  |  | IA 3g | 3g | 33 | applied to the open joint surfaces after all components were cemented into place |  |  | 77.5±17.5 | 14/19 | 63.9±10.6 | 30.6±4.1 | 75.6±17.1 |  |
|  |  | placebo | 0g | 35 |  |  |  | 80.8±14.4 | 13/22 | 68.4±10.4 | 32.7±5.5 | 78.6±14.2 |  |
| Kakar2009 [73] | India | IV 10 mg/kg | < 1g | 12 | before inflation of the tourniquet | yes | yes | NR | 3/9 | 62.4±9.4 | NR | 96.8±17.7 | 7d |
|  |  | placebo | 0g | 12 |  |  |  |  | 4/8 | 66.2±4.8 |  | 92.1±10.8 |  |
|  |  | IV 10 mg/kg | < 1g | 13 | before inflation of the tourniquet | no | yes |  | 4/9 | 63.1±16.8 |  | 154±11.5 |  |
|  |  | placebo | 0g | 13 | before inflation of the tourniquet |  |  |  | 3/10 | 67.2±6.9 |  | 152±17.3 |  |
| Molloy2007 [74] | Northern Ireland | IV 0.5 g twice | 1g | 50 | 5 minutes before deflation of the tourniquet, and a repeat dose three hours later | NR | yes | NR | NR | NR | 29.54±4.9 | 4.9 | 3m |
|  |  | placebo | 0g | 50 |  |  |  |  |  |  | 28.03±4.4 | 4.4 |  |
| Orpen2006 [75] | United Kingdom | IV 15 mg/kg | < 3g | 15 | at the time that cement mixing commenced | yes | yes | NR | 8/7 | 73.0±2.0 | NR | NR | 6m |
|  |  | placebo | 0g | 14 |  |  |  |  | 3/11 | 69.0±2.75 |  |  |  |
| Camarasa2006 [76] | Spain | IV 10 mg/kg twice | < 2g | 35 | before the tourniquet was deflated and 3h later | yes | yes | NR | 9/26 | 73.0±5.75 | NR | 97.0±22. 0 | 3m |
|  |  | placebo | 0g | 60 |  |  |  |  | 32/48 | 72.0±8.25 |  | 102±19.0 |  |
| Good2003 [77] | Sweden | IV 10 mg/kg twice | < 2g | 27 | before release of the tourniquet and repeated after 3h | yes | yes | NR | 6/18 | 72.0±8.50 | 29.0 | NR | 3m |
|  |  | placebo | 0g | 24 |  |  |  |  | 9/18 | 72.0±9.25 | 29.4 |  |  |
| Veien2002 [78] | Sweden | IV 10 mg/kg TXA twice | < 2g | 15 | at conclusion of surgery and again 3h later | yes | yes | 71.2±19.0 | 4/11 | 70.5±9.5 | NR | 71.6±10.4 | 0.5m |
|  |  | placebo | 0g | 15 |  |  |  | 70.3±18.4 | 1/14 | 69.5±9.0 |  | 70.4±21.8 |  |
| Tanaka2001 [79] | Denmark | IV 20 mg/kg TXA | < 2g | 24 | 20 mg/kg of TXA ten minutes before surgery and saline ten minutes before deflation of the tourniquet | yes | yes | 90.0±15.0 | 7/17 | 65.0±2.75 | NR | 110±17.5 | 0.5m |
|  |  | IV 20 mg/kg TXA | < 2g | 22 | saline ten minutes before surgery and 20 mg/kg of TXA ten minutes before deflation of the tourniquet |  |  | 90.0±12.5 | 7/15 | 65.0±2.75 |  | 120±17.5 |  |
|  |  | IV 10 mg/kg TXA twice | < 2g | 27 | 10 mg/kg of TXA ten minutes before surgery and again ten minutes before deflation of the tourniquet |  |  | 90.0±15.0 | 8/19 | 65.0±2.50 | NR | 110±20.0 |  |
|  |  | placebo | 0g | 26 |  |  |  | 95.0±12.5 | 9/17 | 65.0±3.00 |  | 125±17.5 |  |
| Benoni1996 [80] | Sweden | IV 10 mg/kg twice | < 2g | 43 | at a median of 12 minutes before the tourniquet, and repeated 3 h later | yes | yes | 84.0±18.0 | 13/30 | 76.0±7.0 | 26.5 | 96±18 | 1.5m |
|  |  | placebo | 0g | 43 |  |  |  | 84.0±18.0 | 10/33 | 74.0±7.0 | 29.0 | 96±18 |  |
| Hiippala1995 [81] | Finland | IV 15mg/kg | < 2g | 15 | 2 to 5 minutes before deflating the tourniquet | NR | yes | 83.0±18.0 | 2/13 | 70.0±6.5 | 27.4 | 115±18 | 1m |
|  |  | placebo | 0g | 13 |  |  |  | 76.0±16.0 | 3/10 | 70.0±3.75 | 26.9 | 106±17 |  |

NR: not reported.
