# Supplementary material for: Effects of Tranexamic Acid on Hemorrhage Control and Deep Venous Thrombosis Rate After Total Knee Arthroplasty: A Systematic Review and Network Meta-Analysis of Randomized Controlled Trials
Source: Front Pharmacol. 2021 Jul 21;12:639694. doi: 10.3389/fphar.2021.639694 (PMC8335562; doi:10.3389/fphar.2021.639694)
Supplement: Supplementary file 3 [file Image9.pdf]

```
. network sidesplit all, tau

Side     Direct              Indirect             Difference                     tau
         Coef.     Std. Err. Coef.     Std. Err.  Coef.     Std. Err.  P>|z|
A C      -6.609891  113.3371 -46.75157   60.1445   40.14168  128.2062  0.754   149.4056
A E      -135.3175  160.8867 -166.1343  77.27469    30.8168  178.1308  0.863   149.4304
A G      -73.84767  78.29912  32.76622  57.12551  -106.6139   96.9479  0.271   147.8348
A H      -40.55047  76.58649 -89.08041  62.53691   48.52994  98.88951  0.624   149.1501
A I             50  166.5259 -.5309471  121.3743   50.53095  206.0645  0.806   149.3239
A K       -122.134  153.0556 -117.1117  78.57282  -5.022297  172.1526  0.977   149.5595
A L           -373  189.4122 -212.0259  75.21084  -160.9741  203.7981  0.430   148.4391
A N        312.564  37.46202  323.2315  82.63076   -10.6675  91.42306  0.907   149.4338
B C      -70.82005  182.0362 -53.65701  64.33709  -17.16304    193.07  0.929   149.1815
B D *    -149.2026  107.7613 -139.5833  161.9281  -9.619299  194.5433  0.961    149.398
B E      -218.2314  150.5395 -165.2191  84.91124  -53.01229  172.8467  0.759   149.3872
B F *         -310  154.3321 -449.5314  346.3434   139.5314  378.9461  0.713   149.2527
B G      -62.76234  107.7433 -7.215341  64.73041    -55.547  125.6858  0.659   149.3073
B H      -50.16412  166.8346 -92.35044  61.56568   42.18631  177.8479  0.812   149.2996
B I      -103.5346  165.8873  60.70601  128.0997  -164.2406  209.4136  0.433   148.6414
B K      -90.00015   157.195 -148.4747  82.81198   58.47459  177.6741  0.742   149.3552
B N       314.6294  52.03874  244.7267  89.06555   69.90278   103.481  0.499   148.8029
C E      -175.7782  164.3298  -108.431  84.18499  -67.34719  184.6633  0.715   149.2231
C H      -131.0617  152.1128 -14.27069  63.82991   -116.791  164.9609  0.479     148.93
C K      -121.0975   109.966 -50.41217  93.87981  -70.68532  144.5645  0.625   149.2454
C L      -200.3221   153.235 -194.8095  87.00925  -5.512597  176.1776  0.975   149.5271
C N       364.1538  51.86872  314.4228  93.83402     49.731  107.9238  0.645    149.127
D E       -22.2376  150.5389 -40.08418  140.3715   17.84658  205.8375  0.931   149.5225
D F *         -210  154.4928 -70.22099  346.1692   -139.779  378.9832  0.712   149.2528
D H      -51.50013  191.3084  94.45833  108.3242  -145.9585  219.8477  0.507   148.7596
D M *       -244.1  158.2786  261.6349  18911.06  -505.7349  18911.66  0.979   148.1802
D N       614.7384  150.2495  348.4913  111.1248   266.2471  187.7959  0.156   146.5511
E G       26.22583  157.4899  190.1533  80.84029  -163.9274  176.9941  0.354   148.4572
E L      -60.83028  111.7816 -86.93655  113.3092   26.10627  159.1919  0.870   149.3831
E N       459.0121  79.15413  508.5451  117.4099    -49.533  143.9548  0.731   149.2811
G H      -147.2023  88.44477 -23.30816  63.38599  -123.8942  108.8183  0.255   148.0821
G K      -127.0664  158.1528 -110.3898  80.68264  -16.67653  177.4513  0.925   149.4864
G L      -47.52054  153.3179 -281.6274  81.91708   234.1068  174.0604  0.179   147.2134
G N       297.0509  45.69499  387.8912  82.37122  -90.84034  94.53644  0.337   148.4961
H I       -55.5556  166.5799  163.7265   122.519  -219.2821    206.86  0.289    148.001
H J      -83.59991  161.2438  3.567017  108.3852  -87.16693  194.2857  0.654   149.1169
H K      -20.45774  107.8976 -70.28495  95.17175    49.8272  143.8549  0.729   149.3846
H L      -68.82482  152.5146 -192.9655    83.508   124.1406  173.8197  0.475   148.9724
H N        338.689  55.83809  449.4716  67.47624  -110.7826  88.11211  0.209   147.8849
I J         -235.7  162.9759 -10.41187  145.3026  -225.2882  218.3438  0.302   148.0154
J L      -118.5997  175.9795 -151.0489  119.1918   32.44913  212.5452  0.879   149.2994
J N       228.0003  150.7334  496.1049  106.2532  -268.1046  184.4189  0.146   147.0146
K L            -78  153.5374 -133.4821  104.3574   55.48211  185.6454  0.765   149.4684
K N       424.9456   117.524  436.3126  80.90465  -11.36699  145.2113  0.938   149.3948
L N       490.6136  112.5749  578.8377  81.65762  -88.22415  139.1396  0.526   148.9538
```

Supplement Figure 9. Node-splitting approach for total blood loss.

(A: IV TXA ≤ 10mg/kg or 1g once; B: IV TXA ≥ 15mg/kg or 1g once; C: IV TXA ≤ 10mg/kg or 1g twice; D: IV TXA ≥ 15mg/kg or 1g twice; E: IV TXA ≤ 10mg/kg or 1g three times; F: IV TXA ≥ 15mg/kg or 1g three times; G: IA TXA < 2g; H: IA TXA ≥ 2g; I: oral TXA ≤ 2g; J: oral TXA > 2g; K: IV/IV infusion + IA TXA ≤ 3g; L: IV/IV infusion + IA TXA > 3g; M: IV/IV infusion + oral TXA > 3g)
