# Supplementary material for: Effects of Tranexamic Acid on Hemorrhage Control and Deep Venous Thrombosis Rate After Total Knee Arthroplasty: A Systematic Review and Network Meta-Analysis of Randomized Controlled Trials
Source: Front Pharmacol. 2021 Jul 21;12:639694. doi: 10.3389/fphar.2021.639694 (PMC8335562; doi:10.3389/fphar.2021.639694)
Supplement: Supplementary file 4 [file Presentation1.pdf]

Pubmed

1. Search ((((((((((((((tranexamic acid[Title/Abstract]) OR AMCHA[Title/Abstract]) OR trans-4-(Aminomethyl)cyclohexanecarboxylic Acid[Title/Abstract]) OR t-AMCHA[Title/Abstract]) OR AMCA[Title/Abstract]) OR Anvitoff[Title/Abstract]) OR Cyklokapron[Title/Abstract]) OR Ugurol[Title/Abstract]) OR KABI 2161[Title/Abstract]) OR Spotof[Title/Abstract]) OR Transamin[Title/Abstract]) OR Amchafibrin[Title/Abstract]) OR Exacyl[Title/Abstract])) OR "Tranexamic Acid"[Mesh]

2. Search (((((((((((((((((((((((((((((((((total knee arthroplasty[Title/Abstract]) OR Arthroplasties, Replacement, Knee[Title/Abstract]) OR Arthroplasty, Knee Replacement[Title/Abstract]) OR Knee Replacement Arthroplasties[Title/Abstract]) OR Knee Replacement Arthroplasty[Title/Abstract]) OR Replacement Arthroplasties, Knee[Title/Abstract]) OR Knee Arthroplasty, Total[Title/Abstract]) OR Arthroplasty, Total Knee[Title/Abstract]) OR Total Knee Arthroplasty[Title/Abstract]) OR Replacement, Total Knee[Title/Abstract]) OR Total Knee Replacement[Title/Abstract]) OR Knee Replacement, Total[Title/Abstract]) OR Knee Arthroplasty[Title/Abstract]) OR Arthroplasty, Knee[Title/Abstract]) OR Arthroplasties, Knee Replacement[Title/Abstract]) OR Replacement Arthroplasty, Knee[Title/Abstract]) OR Arthroplasty, Replacement, Partial Knee[Title/Abstract]) OR Unicompartmental Knee Arthroplasty[Title/Abstract]) OR Arthroplasty, Unicompartmental Knee[Title/Abstract]) OR Knee Arthroplasty, Unicompartmental[Title/Abstract]) OR Unicondylar Knee Arthroplasty[Title/Abstract]) OR Arthroplasty, Unicondylar Knee[Title/Abstract]) OR Knee Arthroplasty, Unicondylar[Title/Abstract]) OR Partial Knee Arthroplasty[Title/Abstract]) OR Arthroplasty, Partial Knee[Title/Abstract]) OR Unicondylar Knee Replacement[Title/Abstract]) OR Knee Arthroplasty, Partial[Title/Abstract]) OR Knee Replacement, Unicondylar[Title/Abstract]) OR Partial Knee Replacement[Title/Abstract]) OR Knee Replacement, Partial[Title/Abstract]) OR Unicompartmental Knee Replacement[Title/Abstract]) OR Knee Replacement, Unicompartmental[Title/Abstract])) OR "Arthroplasty, Replacement, Knee"[Mesh]

3. randomized controlled trial[Publication Type] OR randomized[Title/Abstract] OR placebo

Embase

1. "tranexamic acid":ab,ti OR "AMCHA":ab,ti OR "trans-4-(Aminomethyl)cyclohexanecarboxylic Acid":ab,ti OR "t-AMCHA":ab,ti OR "AMCA":ab,ti OR "Anvitoff":ab,ti OR "Cyklokapron":ab,ti OR "Ugurol":ab,ti OR "KABI 2161":ab,ti OR "Spotof":ab,ti OR "Transamin":ab,ti OR "Amchafibrin":ab,ti OR "Exacyl":ab,ti

2."total knee arthroplasty":ab,ti OR "Arthroplasties,Replacement, Knee":ab,ti OR "Arthroplasty, Knee Replacement":ab,ti OR "Knee Replacement Arthroplasties":ab,ti OR "Knee Replacement Arthroplasty":ab,ti OR "Replacement Arthroplasties, Knee":ab,ti OR "Knee Arthroplasty, Total":ab,ti OR "Arthroplasty, Total Knee":ab,ti OR "Total Knee Arthroplasty":ab,ti OR "Replacement, Total Knee":ab,ti OR "Total Knee Replacement":ab,ti OR "Knee Replacement, Total":ab,ti OR "Knee Arthroplasty":ab,ti OR "Arthroplasty, Knee":ab,ti OR "Arthroplasties, Knee Replacement":ab,ti OR "Replacement Arthroplasty, Knee":ab,ti OR "Arthroplasty, Replacement, Partial Knee":ab,ti OR "Unicompartmental Knee Arthroplasty":ab,ti OR "Arthroplasty, Unicompartmental Knee":ab,ti OR "Knee Arthroplasty, Unicompartmental":ab,ti OR "Unicondylar Knee Arthroplasty":ab,ti OR "Arthroplasty, Unicondylar Knee":ab,ti OR "Knee Arthroplasty, Unicondylar":ab,ti OR "Partial Knee Arthroplasty":ab,ti OR "Arthroplasty, Partial Knee":ab,ti OR "Knee Arthroplasty, Partial":ab,ti OR "Unicondylar Knee Replacement":ab,ti OR "Knee Replacement, Unicondylar":ab,ti OR "Partial Knee Replacement":ab,ti OR "Knee Replacement, Partial":ab,ti OR "Unicompartmental Knee Replacement":ab,ti OR "Knee Replacement, Unicompartmental":ab,ti

3.“random”:ab,ti OR "placebo":ab,ti OR "double-blind":ab,ti

Cochrane Library

1. "tranexamic acid":ti,ab,kw OR "AMCHA":ti,ab,kw OR "trans-4-(Aminomethyl)cyclohexanecarboxylic Acid":ti,ab,kw OR "t-AMCHA":ti,ab,kw OR "AMCA":ti,ab,kw OR "Anvitoff":ti,ab,kw OR "Cyklokapron":ti,ab,kw OR

"Ugurol":ti,ab,kw OR "KABI 2161":ti,ab,kw OR "Spotof":ti,ab,kw OR "Transamin":ti,ab,kw OR "Amchafibrin":ti,ab,kw OR "Exacyl"

2. "total knee arthroplasty":ti,ab,kw OR "Arthroplasties, Replacement, Knee":ti,ab,kw OR "Arthroplasty, Knee Replacement":ti,ab,kw OR "Knee Replacement Arthroplasties":ti,ab,kw OR "Knee Replacement Arthroplasty":ti,ab,kw OR "Replacement Arthroplasties, Knee":ti,ab,kw OR "Knee Arthroplasty, Total":ti,ab,kw OR "Arthroplasty, Total Knee":ti,ab,kw OR "Total Knee Arthroplasty":ti,ab,kw OR "Replacement, Total Knee":ti,ab,kw OR "Total Knee Replacement":ti,ab,kw OR "Knee Replacement, Total":ti,ab,kw OR "Knee Arthroplasty":ti,ab,kw OR "Arthroplasty, Knee":ti,ab,kw OR "Arthroplasties, Knee Replacement":ti,ab,kw OR "Replacement Arthroplasty, Knee":ti,ab,kw OR "Arthroplasty, Replacement, Partial Knee":ti,ab,kw OR "Unicompartmental Knee Arthroplasty":ti,ab,kw OR "Arthroplasty, Unicompartmental Knee":ti,ab,kw OR "Knee Arthroplasty, Unicompartmental":ti,ab,kw OR "Unicondylar Knee Arthroplasty":ti,ab,kw OR "Arthroplasty, Unicondylar Knee":ti,ab,kw OR "Knee Arthroplasty, Unicondylar":ti,ab,kw OR "Partial Knee Arthroplasty":ti,ab,kw OR "Arthroplasty, Partial Knee":ti,ab,kw OR "Knee Arthroplasty, Partial":ti,ab,kw OR "Unicondylar Knee Replacement":ti,ab,kw OR "Knee Replacement, Unicondylar":ti,ab,kw OR "Partial Knee Replacement":ti,ab,kw OR "Knee Replacement, Partial":ti,ab,kw OR "Unicompartmental Knee Replacement":ti,ab,kw OR "Knee Replacement, Unicompartmental":ti,ab,kw

Web of science

1.TS=(“tranexamic acid” OR “AMCHA” OR “trans-4-(Aminomethyl)cyclohexanecarboxylic Acid” OR “t-AMCHA” OR “AMCA” OR “Anvitoff” OR “Cyklokapron” OR “Ugurol” OR “KABI 2161” OR “Spotof” OR “Transamin” OR “Amchafibrin” OR “Exacyl”)

2.TS=("total knee arthroplasty" OR "Arthroplasties,Replacement, Knee" OR "Arthroplasty, Knee Replacement" OR "Knee Replacement Arthroplasties" OR "Knee Replacement Arthroplasty" OR "Replacement Arthroplasties, Knee" OR "Knee Arthroplasty, Total" OR "Arthroplasty, Total Knee" OR "Total Knee Arthroplasty" OR

"Replacement, Total Knee" OR "Total Knee Replacement" OR "Knee Replacement, Total" OR "Knee Arthroplasty" OR "Arthroplasty, Knee" OR "Arthroplasties, Knee Replacement" OR "Replacement Arthroplasty, Knee" OR "Arthroplasty, Replacement, Partial Knee" OR "Unicompartmental Knee Arthroplasty" OR "Arthroplasty, Unicompartmental Knee" OR "Knee Arthroplasty, Unicompartmental" OR "Unicondylar Knee Arthroplasty" OR "Arthroplasty, Unicondylar Knee" OR "Knee Arthroplasty, Unicondylar" OR "Partial Knee Arthroplasty" OR "Arthroplasty, Partial Knee" OR "Knee Arthroplasty, Partial" OR "Unicondylar Knee Replacement" OR "Knee Replacement, Unicondylar" OR "Partial Knee Replacement" OR "Knee Replacement, Partial" OR "Unicompartmental Knee Replacement" OR "Knee Replacement, Unicompartmental")

3.TS=(random* controlled trial OR random* OR placebo)

1. Wang, H.Y., Wang, L., Luo, Z.Y., Wang, D., Tang, X., Zhou, Z.K., and Pei, F.X. (2019). Intravenous and subsequent long-term oral tranexamic acid in enhanced-recovery primary total knee arthroplasty without the application of a tourniquet: A randomized placebo-controlled trial. BMC Musculoskelet. Disord. 20.

2. Tzatzairis, T., Drosos, G.I., Vogiatzaki, T., Tilkeridis, K., Ververidis, A., and Kazakos, K. (2019). Multiple intravenous tranexamic acid doses in total knee arthroplasty without tourniquet: a randomized controlled study. Arch. Orthop. Trauma Surg. 139, 859-868.

3. Tang, Y., Wen, Y., Li, W., Li, H., Yang, Y., and Liu, Y. (2019). The efficacy and safety of multiple doses of oral tranexamic acid on blood loss, inflammatory and fibrinolysis response following total knee arthroplasty: A randomized controlled trial. International Journal of Surgery 65, 45-51.

4. Tammachote, N., Raphiphan, R., and Kanitnate, S. (2019). High-dose (3g) topical tranexamic acid has higher potency in reducing blood loss after total knee arthroplasty compared with low dose (500mg): a double-blind randomized controlled trial. European journal of orthopaedic surgery & traumatology : orthopedie traumatologie 29, 1729-1735.

5. Nambiar, H.R., and Kamath, S.S. (2019). Comparison of efficacy of tranexamic acid 10 mg/kg and 15 mg/kg in reducing bleeding and transfusions in total knee arthroplasty. Asian journal of pharmaceutical and clinical research 12, 535-538.
6. King, L., Randle, R., Dare, W., and Bernaitis, N. (2019). Comparison of oral vs. combined topical/intravenous/oral tranexamic acid in the prevention of blood loss in total knee arthroplasty: A randomised clinical trial. Orthopaedics & Traumatology-Surgery & Research 105, 1073-1077.
7. Wang, D., Wang, H.Y., Cao, C., Li, L.L., Meng, W.K., Pei, F.X., Li, D.H., Zhou, Z.K., and Zeng, W.N. (2018). Tranexamic acid in primary total knee arthroplasty without tourniquet: a randomized, controlled trial of oral versus intravenous versus topical administration. Sci. Rep. 8, 13579.
8. Wang, D., Zhu, H., Meng, W.K., Wang, H.Y., Luo, Z.Y., Pei, F.X., Li, Q., and Zhou, Z.K. (2018). Comparison of oral versus intra-articular tranexamic acid in enhanced-recovery primary total knee arthroplasty without tourniquet application: a randomized controlled trial. BMC Musculoskelet. Disord. 19, 85.
9. Takuya Iseki, S.T., Motohiro Wakui, Shinichi Yoshiya (2018). Intravenous tranexamic acid only versus combined intravenous and intra-articular tranexamic acid for perioperative blood loss in patients undergoing total knee arthroplasty. European journal of orthopaedic surgery & traumatology : orthopedie traumatologie, 1-6.
10. Mehta, N., Goel, N., Goyal, A., Joshi, D., and Chaudhary, D. (2018). A prospective comparative study between intravenous and intraarticular tranexamic acid administration in decreasing the perioperative blood loss in total knee arthroplasty. Journal of arthroscopy and joint surgery (no pagination).
11. Almeida, M.D.C.d., Albuquerque, R.P.E., Palhares, G.M., Almeida, J.P.C.d., Barretto, J.M., and Cavanellas, N. (2018). Evaluation of the use of tranexamic acid in total knee arthroplasty. Revista brasileira de ortopedia 53, 761-767.
12. Adravanti, P., Di Salvo, E., Calafiore, G., Vasta, S., Ampollini, A., and Rosa, M.A. (2018). A prospective, randomized, comparative study of intravenous alone and combined intravenous and intraarticular administration of tranexamic acid in primary total knee replacement. Arthroplasty today 4, 85-88.

13. Kwok Pui Pui , H.K.K., Yang Isaac Bruce, Sha Wai Leung, Wong Hok Leung, Chow Yuk Yin (2018). Effect of topical tranexamic acid on reducing blood loss in primary total knee arthroplasty in Southern Chinese population. Journal of orthopaedics, trauma and rehabilitation 25.
14. George, J., Eachempati, K.K., Subramanyam, K.N., and Reddy, A.V.G. (2018). The comparative efficacy and safety of topical and intravenous tranexamic acid for reducing perioperative blood loss in Total knee arthroplasty- A randomized controlled non-inferiority trial. Knee 25, 185-191.
15. Yuan, X., Li, B., Wang, Q., and Zhang, X. (2017). Comparison of 3 Routes of Administration of Tranexamic Acid on Primary Unilateral Total Knee Arthroplasty: A Prospective, Randomized, Controlled Study. J. Arthroplasty 32, 2738-2743.
16. Liu, W., Yang, C., Huang, X., and Liu, R. (2018). Tranexamic Acid Reduces Occult Blood Loss, Blood Transfusion, and Improves Recovery of Knee Function after Total Knee Arthroplasty: A Comparative Study. J. Knee Surg. 31, 239-246.
17. Yen, S.-H., Lin, P.-C., Chen, B., Huang, C.-C., and Wang, J.-W. (2017). Topical Tranexamic Acid Reduces Blood Loss in Minimally Invasive Total Knee Arthroplasty Receiving Rivaroxaban. Biomed Research International.
18. Wang, J.-W., Chen, B., Lin, P.-C., Yen, S.-H., Huang, C.-C., and Kuo, F.-C. (2017). The Efficacy of Combined Use of Rivaroxaban and Tranexamic Acid on Blood Conservation in Minimally Invasive Total Knee Arthroplasty a Double-Blind Randomized, Controlled Trial. J. Arthroplasty 32, 801-806.
19. Wang, J., Wang, Q , Zhang, X , and Wang, Q. (2017). Intra-articular Application is More Effective Than Intravenous Application of Tranexamic Acid in Total Knee Arthroplasty: A Prospective Randomized Controlled Trial. J. Arthroplasty 32, 3385-3389.
20. Ugurlu, M., Aksekili, M.A.E., Caglar, C., Yuksel, K., Sahin, E., and Akyol, M. (2017). Effect of Topical and Intravenously Applied Tranexamic Acid Compared to Control Group on Bleeding in Primary Unilateral Total Knee Arthroplasty. J. Knee Surg. 30, 152-157.
21. Sun, Q., Yu, X., Wu, J., Ge, W., Cai, M., and Li, S. (2017). Efficacy of a Single

Dose and an Additional Dose of Tranexamic Acid in Reduction of Blood Loss in Total Knee Arthroplasty. J. Arthroplasty 32, 2108-2112.
22. Stowers, M.D.J., Aoina, J., Vane, A., Poutawera, V., Hill, A.G., and Munro, J.T. (2017). Tranexamic Acid in Knee Surgery Study-A Multicentered, Randomized, Controlled Trial. J. Arthroplasty 32, 3379-3384.
23. Song, E.-K., Seon, J.-K., Prakash, J., Seol, Y.-J., Park, Y.J., and Jin, C. (2017). Combined Administration of IV and Topical Tranexamic Acid is Not Superior to Either Individually in Primary Navigated TKA. J. Arthroplasty 32, 37-42.
24. Prakash, J., Seon, J.K., Park, Y.J., Jin, C., and Song, E.K. (2017). A randomized control trial to evaluate the effectiveness of intravenous, intra-articular and topical wash regimes of tranexamic acid in primary total knee arthroplasty. J. Orthop. Surg. (Hong Kong) 25, 2309499017693529.
25. Lee, S.Y., Chong, S., Balasubramanian, D., Na, Y.G., and Kim, T.K. (2017). What is the Ideal Route of Administration of Tranexamic Acid in TKA? A Randomized Controlled Trial. Clin. Orthop. Relat. Res. 475, 1987-1996.
26. Lee, Q.J., Ching, W.Y., and Wong, Y.C. (2017). Blood Sparing Efficacy of Oral Tranexamic Acid in Primary Total Knee Arthroplasty: A Randomized Controlled Trial. Knee surgery & related research 29, 57-62.
27. Huang, Z., Xie, X., Li, L., Huang, Q., Ma, J., Shen, B., Kraus, V.B., and Pei, F. (2017). Intravenous and Topical Tranexamic Acid Alone Are Superior to Tourniquet Use for Primary Total Knee Arthroplasty A Prospective, Randomized Controlled Trial. Journal of Bone and Joint Surgery-American Volume 99, 2053-2061.
28. Xu Jian, H.C.-z., Tian Shao-qi (2016). Intra-articular and intravenous injection of tranexamic acid effectively reduces blood loss after total knee arthroplasty.
29. Zekcer, A., Del Priori, R., Tieppo, C., da Silva, R.S., and Severino, N.R. (2016). Topical vs. intravenous administration of tranexamic acid in knee arthroplasty and prevalence of deep venous thrombosis: a randomized clinical trial. Jornal vascular brasileiro 15, 120-125.
30. Volquind, D., Zardo, R.A., Winkler, B.C., Londero, B.B., Zanelatto, N., and Leichtweis, G.P. (2016). Use of tranexamic acid in primary total knee replacement:

effects on perioperative blood loss. Rev. Bras. Anestesiol. 66, 254-258.

31. Tzatzairis, T.K., Drosos, G.I., Kotsios, S.E., Ververidis, A.N., Vogiatzaki, T.D., and Kazakos, K.I. (2016). Intravenous vs Topical Tranexamic Acid in Total Knee Arthroplasty Without Tourniquet Application: A Randomized Controlled Study. J. Arthroplasty 31, 2465-2470.

32. Seviciu, A., Gross, I., Fathima, S., and Walsh, S.M. (2016). Effects of tranexamic acid and bipolar sealer alone or in combination in primary total knee arthroplasty: a prospective, randomized, controlled trial. Arthroplasty today 2, 77-82.

33. Nielsen, C.S., Jans, O., Orsnes, T., Foss, N.B., Troelsen, A., and Husted, H. (2016). Combined Intra-Articular and Intravenous Tranexamic Acid Reduces Blood Loss in Total Knee Arthroplasty: A Randomized, Double-Blind, Placebo-Controlled Trial. J. Bone Joint Surg. Am. 98, 835-841.

34. Keyhani, S., Esmailiejah, A.A., Abbasian, M.R., and Safdari, F. (2016). Which Route of Tranexamic Acid Administration is More Effective to Reduce Blood Loss Following Total Knee Arthroplasty? The archives of bone and joint surgery 4, 65-69.

35. Fillingham, Y.A., Kayupov, E., Plummer, D.R., Moric, M., Gerlinger, T.L., and Della Valle, C.J. (2016). The James A. Rand Young Investigator's Award: A Randomized Controlled Trial of Oral and Intravenous Tranexamic Acid in Total Knee Arthroplasty: The Same Efficacy at Lower Cost? J. Arthroplasty 31, 26-30.

36. Guzel, Y., Gurcan, O.T., Golge, U.H., Dulgeroglu, T.C., and Metineren, H. (2016). Topical tranexamic acid versus autotransfusion after total knee arthroplasty. Journal of Orthopaedic Surgery 24, 179-182.

37. Drosos, G.I., Ververidis, A., Valkanis, C., Tripsianis, G., Stavroulakis, E., Vogiatzaki, T., and Kazakos, K. (2016). A randomized comparative study of topical versus intravenous tranexamic acid administration in enhanced recovery after surgery (ERAS) total knee replacement. Journal of orthopaedics 13, 127-131.

38. Chen, X., Cao, X., Yang, C., Guo, K., Zhu, Q., and Zhu, J. (2016). Effectiveness and Safety of Fixed-Dose Tranexamic Acid in Simultaneous Bilateral Total Knee Arthroplasty: A Randomized Double-Blind Controlled Trial. J. Arthroplasty 31, 2471-2475.

39. Castro-Menendez, M., Pena-Paz, S., Rocha-Garcia, F., Rodriguez-Casas, N., Huici-Izco, R., and Montero-Vieites, A. (2016). Efficacy of 2 grammes of intravenous transexamic acid in the reduction of post-surgical bleeding after total hip and knee replacement. Revista espanola de cirugia ortopedicay traumatologia 60, 315-324.
40. Yang, Y., Lv, Y.M., Ding, P.J., Li, J., and Ying-Ze, Z. (2015). The reduction in blood loss with intra-articular injection of tranexamic acid in unilateral total knee arthroplasty without operative drains: a randomized controlled trial. Eur. J. Orthop. Surg. Traumatol. 25, 135-139.
41. Wang, G., Wang, D., Wang, B., Lin, Y., and Sun, S. (2015). Efficacy and safety evaluation of intra-articular injection of tranexamic acid in total knee arthroplasty operation with temporarily drainage close. Int. J. Clin. Exp. Med. 8, 14328-14334.
42. Wang, C.G., Sun, Z.H., Liu, J., Cao, J.G., and Li, Z.J. (2015). Safety and efficacy of intra-articular tranexamic acid injection without drainage on blood loss in total knee arthroplasty: A randomized clinical trial. Int. J. Surg. 20, 1-7.
43. Shinde, A., Sobti, A., Maniar, S., Mishra, A., Gite, R., and Shetty, V. (2015). Tranexamic acid reduces blood loss and need of blood transfusion in total knee arthroplasty: A prospective, randomized, double-blind study in Indian population. Asian J. Transfus. Sci. 9, 168-172.
44. Shen, P.F., Hou, W.L., Chen, J.B., Wang, B., and Qu, Y.X. (2015). Effectiveness and safety of tranexamic acid for total knee arthroplasty: a prospective randomized controlled trial. Med. Sci. Monit. 21, 576-581.
45. Oztas, S., Ozturk, A., Akalin, Y., Sahin, N., Ozkan, Y., Otuzbir, A., and Avcu, B. (2015). The effect of local and systemic application of tranexamic acid on the amount of blood loss and allogeneic blood transfusion after total knee replacement. Acta Orthop. Belg. 81, 698-707.
46. Motififard, M., Tahririan, M.A., Saneie, M., Badiei, S., and Nemati, A. (2015). Low Dose Perioperative Intravenous Tranexamic Acid in Patients Undergoing Total Knee Arthroplasty: A Double-Blind Randomized Placebo Controlled Clinical Trial. Journal of blood transfusion 2015, 948304-948304.
47. Lin, S.Y., Chen, C.H., Fu, Y.C., Huang, P.J., Chang, J.K., and Huang, H.T. (2015).

The efficacy of combined use of intraarticular and intravenous tranexamic acid on reducing blood loss and transfusion rate in total knee arthroplasty. J. Arthroplasty 30, 776-780.

48. Kundu, R., Das, A., Basunia, S.R., Bhattacharyya, T., Chattopadhyay, S., and Mukherjee, A. (2015). Does a single loading dose of tranexamic acid reduce perioperative blood loss and transfusion requirements after total knee replacement surgery? A randomized, controlled trial. J. Nat. Sci. Biol. Med. 6, 94-99.

49. Karaaslan, F., Karaoğlu, S., Mermerkaya, M.U., and Baktir, A. (2015). Reducing blood loss in simultaneous bilateral total knee arthroplasty: combined intravenous-intra-articular tranexamic acid administration. A prospective randomized controlled trial. The knee 22, 131-135.

50. Carvalho, L.H., Jr., Frois Temponi, E., Machado Soares, L.F., Goncalves, M.B.J., Paiva Costa, L., and Tavares de Souza, M.L.A. (2015). Bleeding reduction after topical application of tranexamic acid together with Betadine solution in total knee arthroplasty. A randomised controlled study. Orthopaedics & Traumatology-Surgery & Research 101, 83-87.

51. Aguilera, X., Martinez-Zapata, M.J., Hinarejos, P., Jordan, M., Leal, J., Gonzalez, J.C., Monllau, J.C., Celaya, F., Rodriguez-Arias, A., Fernandez, J.A., et al. (2015). Topical and intravenous tranexamic acid reduce blood loss compared to routine hemostasis in total knee arthroplasty: a multicenter, randomized, controlled trial. Arch. Orthop. Trauma Surg. 135, 1017-1025.

52. Sarzaeem, M.M., Razi, M., Kazemian, G., Moghaddam, M.E., Rasi, A.M., and Karimi, M. (2014). Comparing Efficacy of Three Methods of Tranexamic Acid Administration in Reducing Hemoglobin Drop Following Total Knee Arthroplasty. J. Arthroplasty 29, 1521-1524.

53. Patel, J.N., Spanyer, J.M., Smith, L.S., Huang, J., Yakkanti, M.R., and Malkani, A.L. (2014). Comparison of Intravenous versus Topical Tranexamic Acid in Total Knee Arthroplasty: A Prospective Randomized Study. J. Arthroplasty 29, 1528-1531.

54. Levine, B.R., Haughom, B.D., Belkin, M.N., and Goldstein, Z.H. (2014). Weighted Versus Uniform Dose of Tranexamic Acid in Patients Undergoing Primary, Elective

Knee Arthroplasty: A Prospective Randomized Controlled Trial. J. Arthroplasty 29, 186-188.
55. Kim, T.K., Chang, C.B., Kang, Y.G., Seo, E.S., Lee, J.H., Yun, J.H., and Lee, S.H. (2014). Clinical value of tranexamic acid in unilateral and simultaneous bilateral TKAs under a contemporary blood-saving protocol: a randomized controlled trial. Knee Surgery Sports Traumatology Arthroscopy 22, 1870-1878.
56. Huang, Z., Ma, J., Shen, B., and Pei, F. (2014). Combination of Intravenous and Topical Application of Tranexamic Acid in Primary Total Knee Arthroplasty: A Prospective Randomized Controlled Trial. J. Arthroplasty 29, 2342-2346.
57. Martin, J.G., Cassatt, K.B., Kincaid-Cinnamon, K.A., Westendorf, D.S., Garton, A.S., and Lemke, J.H. (2014). Topical Administration of Tranexamic Acid in Primary Total Hip and Total Knee Arthroplasty. J. Arthroplasty 29, 889-894.
58. Bidolegui, F., Arce, G., Lugones, A., Pereira, S., and Vindver, G. (2014). Tranexamic Acid Reduces Blood Loss and Transfusion in Patients Undergoing Total Knee Arthroplasty without Tourniquet: A Prospective Randomized Controlled Trial. The open orthopaedics journal 8, 250-254.
59. Antinolfi, P., Innocenti, B., Caraffa, A., Peretti, G., and Cerulli, G. (2014). Post-operative blood loss in total knee arthroplasty: knee flexion versus pharmacological techniques. Knee Surgery Sports Traumatology Arthroscopy 22, 2756-2762.
60. Gomez-Barrena, E., Ortega-Andreu, M., Padilla-Eguiluz, N.G., Pérez-Chrzanowska, H., and Figueredo-Zalve, R. (2014). Topical intra-articular compared with intravenous tranexamic acid to reduce blood loss in primary total knee replacement: a double-blind, randomized, controlled, noninferiority clinical trial. Journal of bone and joint surgery. American volume 96, 1937-1944.
61. Seo, J.-G., Moon, Y.-W., Park, S.-H., Kim, S.-M., and Ko, K.-R. (2013). The comparative efficacies of intra-articular and IV tranexamic acid for reducing blood loss during total knee arthroplasty. Knee Surgery Sports Traumatology Arthroscopy 21, 1869-1874.
62. Sa-ngasoongsong, P., Wongsak, S., Chanplakorn, P., Woratanarat, P., Wechmongkolgorn, S., Wibulpolprasert, B., Mulpruek, P., and Kawinwonggowit, V.

(2013). Efficacy of low-dose intra-articular tranexamic acid in total knee replacement; a prospective triple-blinded randomized controlled trial. BMC Musculoskelet. Disord. 14.

63. Georgiadis, A.G., Muh, S.J., Silverton, C.D., Weir, R.M., and Laker, M.W. (2013). A Prospective Double-Blind Placebo Controlled Trial of Topical Tranexamic Acid in Total Knee Arthroplasty. J. Arthroplasty 28, 78-82.

64. Lee, S.H., Cho, K.-Y., Khurana, S., and Kim, K.-I. (2013). Less blood loss under concomitant administration of tranexamic acid and indirect factor Xa inhibitor following total knee arthroplasty: a prospective randomized controlled trial. Knee Surgery Sports Traumatology Arthroscopy 21, 2611-2617.

65. Alshryda, S., Mason, J., Vaghela, M., Sarda, P., Nargol, A., Maheswaran, S., Tulloch, C., Anand, S., Logishetty, R., Stothart, B., et al. (2013). Topical (intra-articular) tranexamic acid reduces blood loss and transfusion rates following total knee replacement: a randomized controlled trial (TRANX-K). Journal of bone and joint surgery. American volume 95, 1961-1968.

66. Aguilera, X., Martinez-Zapata, M.J., Bosch, A., Urrútia, G., González, J.C., Jordan, M., Gich, I., Maymó, R.M., Martínez, N., Monllau, J.C., et al. (2013). Efficacy and safety of fibrin glue and tranexamic acid to prevent postoperative blood loss in total knee arthroplasty: a randomized controlled clinical trial. Journal of bone and joint surgery. American volume 95, 2001-2007.

67. McConnell, J.S., Shewale, S., Munro, N.A., Shah, K., Deakin, A.H., and Kinninmonth, A.W.G. (2012). Reducing blood loss in primary knee arthroplasty: A prospective randomised controlled trial of tranexamic acid and fibrin spray. Knee 19, 295-298.

68. Maniar, R.N., Kumar, G., Singhi, T., Nayak, R.M., and Maniar, P.R. (2012). Most Effective Regimen of Tranexamic Acid in Knee Arthroplasty: A Prospective Randomized Controlled Study in 240 Patients. Clin. Orthop. Relat. Res. 470, 2605-2612.

69. Lin, P.C., Hsu, C.H., Huang, C.C., Chen, W.S., and Wang, J.W. (2012). The blood-saving effect of tranexamic acid in minimally invasive total knee replacement: Is an

additional pre-operative injection effective? Journal of Bone and Joint Surgery - Series B 94 B, 932-936.

70. Sa-Ngasoongsong, P., Channoom, T., Kawinwonggowit, V., Woratanarat, P., Chanplakorn, P., Wibulpolprasert, B., Wongsak, S., Udomsubpayakul, U., Wechmongkolgorn, S., and Lekpittaya, N. (2011). Postoperative blood loss reduction in computer-assisted surgery total knee replacement by low dose intra-articular tranexamic acid injection together with 2-hour clamp drain: a prospective triple-blinded randomized controlled trial. Orthop. Rev. (Pavia) 3, e12-e12.

71. MacGillivray, R.G., Tarabichi, S.B., Hawari, M.F., and Raoof, N.T. (2011). Tranexamic acid to reduce blood loss after bilateral total knee arthroplasty: a prospective, randomized double blind study. J. Arthroplasty 26, 24-28.

72. Wong, J., Abrishami, A., El Beheiry, H., Mahomed, N.N., Davey, J.R., Gandhi, R., Syed, K.A., Hasan, S.M.O., De Silva, Y., and Chung, F. (2010). Topical application of tranexamic acid reduces postoperative blood loss in total knee arthroplasty: A randomized, controlled trial. Journal of Bone and Joint Surgery - Series A 92, 2503-2513.

73. Kakar, P.N., Gupta, N., Govil, P., and Shah, V. (2009). Efficacy and Safety of Tranexamic Acid in Control of Bleeding Following TKR: A Randomized Clinical Trial. Indian J. Anaesth. 53, 667-671.

74. Molloy, D.O., Archbold, H.A.P., Ogonda, L., McConway, J., Wilson, R.K., and Beverland, D.E. (2007). Comparison of topical fibrin spray and tranexamic acid on blood loss after total knee replacement: a prospective, randomised controlled trial. Journal of bone & joint surgery - british volume 89.

75. Orpen, N.M., Little, C., Walker, G., and Crawfurd, E.J.P. (2006). Tranexamic acid reduces early post-operative blood loss after total knee arthroplasty: A prospective randomised controlled trial of 29 patients. Knee 13, 106-110.

76. Camarasa, M.A., Ollé, G., Serra-Prat, M., Martín, A., Sánchez, M., Ricós, P., Pérez, A., and Opisso, L. (2006). Efficacy of aminocaproic, tranexamic acids in the control of bleeding during total knee replacement: A randomized clinical trial. Br. J. Anaesth. 96, 576-582.

77. Good, L., Peterson, E., and Lisander, B. (2003). Tranexamic acid decreases external blood loss but not hidden blood loss in total knee replacement. Br. J. Anaesth. 90, 596-599.
78. Veien, M., Sørensen, J.V., Madsen, F., and Juelsgaard, P. (2002). Tranexamic acid given intraoperatively reduces blood loss after total knee replacement: a randomized, controlled study. Acta Anaesthesiol. Scand. 46, 1206-1211.
79. Tanaka, N., Sakahashi, H., Sato, E., Hirose, K., Ishima, T., and Ishii, S. (2001). Timing of the administration of tranexamic acid for maximum reduction in blood loss in arthroplasty of the knee. Journal of bone & joint surgery - british volume 83, 702-705.
80. Benoni, G., and Fredin, H. (1996). Fibrinolytic inhibition with tranexamic acid reduces blood loss and blood transfusion after knee arthroplasty: a prospective, randomised, double-blind study of 86 patients. J. Bone Joint Surg. Br. 78, 434-440.
81. Hiippala, S., Strid, L., Wennerstrand, M., Arvela, V., Mäntylä, S., Ylinen, J., and Niemelä, H. (1995). Tranexamic acid (Cyklokapron) reduces perioperative blood loss associated with total knee arthroplasty. Br. J. Anaesth. 74, 534-537.
