# Supplementary material for: Effects of Tranexamic Acid on Hemorrhage Control and Deep Venous Thrombosis Rate After Total Knee Arthroplasty: A Systematic Review and Network Meta-Analysis of Randomized Controlled Trials
Source: Front Pharmacol. 2021 Jul 21;12:639694. doi: 10.3389/fphar.2021.639694 (PMC8335562; doi:10.3389/fphar.2021.639694)
Supplement: Supplementary file 5 [file Image10.pdf]

```
. network sidesplit all, tau

Side     Direct               Indirect             Difference                     tau
         Coef.     Std. Err.  Coef.     Std. Err.  Coef.     Std. Err.  P>|z|
A B       .0099594  .4266364  -.2028625  .4644932   .2128219  .6305122  0.736    4.41e-09
A C       1.317029  1.595263   .1176899  .4353802   1.199339  1.659411  0.470    5.40e-10
A G      -.0088029  .8214252   .0071436  .4847593  -.0159465  .9537982  0.987    1.79e-09
A H       -.340978  .8517476  -.3934316  .4239692   .0524536  .9492243  0.956    1.19e-10
A K       .7849568  2.019567  -.3287545  .8774745   1.113711  2.200904  0.613    1.32e-09
A L       7.72e-11  2.016327    .350962   .953937   -.350962  2.230598  0.875    2.05e-09
A N       .0620272  .2545446   .3004322  .6497168   -.238405  .6966763  0.732    7.57e-10
B D *     .4774955  .9287455   .5147728  .6615884  -.0372773  1.152049  0.974    1.34e-09
B E       2.19e-11     1.174   .1785239   .792241  -.1785239  1.416306  0.900    9.45e-10
B F *     1.37e-11  2.008247    1.08035   3.65001   -1.08035  4.166009  0.795    3.73e-10
B G       1.127547  1.079984  -.1515498  .4981774   1.279097  1.215359  0.293    2.44e-09
B H      -.6331802  1.072884  -.2416316  .4288045  -.3915486  1.155831  0.735    4.23e-11
B I      -1.021057  1.605046   1.466871  1.617258  -2.487928  2.148575  0.247    3.94e-10
B K      -1.109481  1.639635   .2651377  .9215212  -1.374618   1.88085  0.465    5.10e-10
B N       .2564978  .2935447   -.163512  .6128111   .4200098  .6724285  0.532    9.76e-12
C D       4.02e-11  1.198289   .2739793  .6597748  -.2739793  1.367918  0.841    2.19e-10
C H      -.4340467  1.053097  -.6225064  .5027525   .1884597   1.16695  0.872    1.79e-09
C K       .0024751  1.418389  -.5385668  1.009308   .5410419  1.740847  0.756    1.13e-09
C L      -.0211939  2.005285   .1082031  1.002314   -.129397  2.241838  0.954    2.21e-09
C N      -.1234398  .3981168   -.055452  .8685926  -.0679878     .9702  0.944    1.69e-10
D E      -.5792987  1.046721  -.2059048  .9765288   -.373394  1.419121  0.792    9.40e-12
D F *     2.31e-12  2.008247  -1.080364  3.650014   1.080364  4.166013  0.795    7.37e-10
D H      -.4349281  1.053799  -.9666043  .6950592   .5316762  1.311244  0.685    7.75e-10
D J      -.5441119  1.031848   .1586647   1.07833  -.7027767  1.470651  0.633    1.20e-10
D M *     .5108256  .7547827  -.8939807  251.7444   1.404806  251.7461  0.996    3.16e-09
D N       -.853059  .7004426   .5170037  .9201864  -1.370063  1.261971  0.278    4.81e-09
E H       8.50e-11  2.009877  -.4735777  .7303917   .4735777  2.138475  0.825    4.06e-10
E L       6.30e-11  2.009877   .3295803  1.119592  -.3295803  2.300672  0.886    1.75e-10
E N      -.0368612   .743336   .3364053  1.335413  -.3732666  1.584029  0.814    4.41e-10
G H      -.2231742  .8656172  -.4511675  .5481885   .2279932  1.022394  0.824    1.40e-09
G K       3.13e-11  2.012308  -.1886171  .9323157   .1886171  2.217791  0.932    3.68e-10
G N       .1140592  .4162522  -.0037093  .8190561   .1177685  .9114611  0.897    7.36e-10
H I       2.49e-11  2.008247   .8018198  1.527424  -.8018198   2.52311  0.751    1.30e-09
H J       .9594969  1.121308   .2564474  1.055183   .7030495  1.637707  0.668    1.51e-09
H K       .2025413  1.423877   .2448515  1.002709  -.0423102  1.741013  0.981    9.23e-10
H L       -.021164  2.005285   .8353864  .9814196  -.8565505  2.232575  0.701    2.53e-10
H N       .5556938  .3884196   .2988758  .5762824    .256818   .687243  0.709    5.73e-10
I J      -1.118615  1.645194   1.440498  1.751211  -2.559113  2.402796  0.287    4.96e-11
J L      -.1112256   2.01849   .1439846  1.158414  -.2552103  2.327279  0.913    1.92e-09
J N      -.3739535  .9474231   .1820672  1.005819  -.5560207  1.381769  0.687    5.40e-10
K L       3.73e-11  2.005229   .6261513  1.309097  -.6261513  2.394719  0.794    9.01e-10
K N       .0273798  1.428295   .3399837  .9383683  -.3126039  1.709056  0.855    5.60e-10
L N      -1.098609  1.648041   .1293238  .9838332  -1.227933   1.91937  0.522    1.93e-09
```

Supplement Figure 10. Node-splitting approach for DVT rate.

(A: IV TXA ≤ 10mg/kg or 1g once; B: IV TXA ≥ 15mg/kg or 1g once; C: IV TXA ≤ 10mg/kg or 1g twice; D: IV TXA ≥ 15mg/kg or 1g twice; E: IV TXA ≤ 10mg/kg or 1g three times; F: IV TXA ≥ 15mg/kg or 1g three times; G: IA TXA < 2g; H: IA TXA ≥ 2g; I: oral TXA ≤ 2g; J: oral TXA > 2g; K: IV/IV infusion + IA TXA ≤ 3g; L: IV/IV infusion + IA TXA > 3g; M: IV/IV infusion + oral TXA > 3g)
