# Supplementary material for: Effects of Tranexamic Acid on Hemorrhage Control and Deep Venous Thrombosis Rate After Total Knee Arthroplasty: A Systematic Review and Network Meta-Analysis of Randomized Controlled Trials
Source: Front Pharmacol. 2021 Jul 21;12:639694. doi: 10.3389/fphar.2021.639694 (PMC8335562; doi:10.3389/fphar.2021.639694)
Supplement: Supplementary file 7 [file Image8.pdf]

```
. network meta i
Command is: mvmeta _y _S  , bscovariance(exch 0.5) longparm suppress(uv mm) eq(_y_B: des_ABN, _y_C: des_CDN des_CN, _y_D: des_BDF de
> s_CDN des_DH des_DHJN des_DN, _y_E: des_EHN des_EL des_EN, _y_G: des_AGN des_BGN des_GHN des_GKN des_GN, _y_H: des_AHKN des_AHN de
> s_BHI des_BHN des_CH des_CHKL des_DHJN des_EHN des_GH des_GHN des_HN, _y_J: des_HJ des_IJ des_JL des_JN, _y_K: des_BK des_CHKL des
> _CK des_GKN, _y_L: des_CHKL des_LN, _y_N: des_ACN des_AGN des_AHKN des_AHN des_AN des_BDEN des_BGN des_BHN des_BN) vars(_y_B _y_C
> _y_D _y_E _y_F _y_G _y_H _y_I _y_J _y_K _y_L _y_M _y_N)
Note: using method reml
Note: regressing _y_B on des_ABN
Note: regressing _y_C on des_CDN des_CN
Note: regressing _y_D on des_BDF des_CDN des_DH des_DHJN des_DN
Note: regressing _y_E on des_EHN des_EL des_EN
Note: regressing _y_F on (nothing)
Note: regressing _y_G on des_AGN des_BGN des_GHN des_GKN des_GN
Note: regressing _y_H on des_AHKN des_AHN des_BHI des_BHN des_CH des_CHKL des_DHJN des_EHN des_GH des_GHN des_HN
Note: regressing _y_I on (nothing)
Note: regressing _y_J on des_HJ des_IJ des_JL des_JN
Note: regressing _y_K on des_BK des_CHKL des_CK des_GKN
Note: regressing _y_L on des_CHKL des_LN
Note: regressing _y_M on (nothing)
Note: regressing _y_N on des_ACN des_AGN des_AHKN des_AHN des_AN des_BDEN des_BGN des_BHN des_BN
Note: 73 observations on 13 variables
Note: variance-covariance matrix is proportional to .5*I(13)+.5*J(13,13,1)

initial:       log likelihood = -385.61725
rescale:       log likelihood = -383.03627
rescale eq:    log likelihood = -380.09365
Iteration 0:   log likelihood = -380.09365
Iteration 1:   log likelihood = -379.92925
Iteration 2:   log likelihood = -379.92919
Iteration 3:   log likelihood = -379.92919

Multivariate meta-analysis
Variance-covariance matrix = proportional .5*I(13)+.5*J(13,13,1)
Method = reml                                   Number of dimensions    =    13
Restricted log likelihood = -379.92919          Number of observations  =    73

------------------------------------------------------------------------------
             |      Coef.   Std. Err.      z    P>|z|     [95% Conf. Interval]
-------------+----------------------------------------------------------------
_y_B         |
     des_ABN |   .0097674   2.055771     0.00   0.996    -4.019469    4.039004
       _cons |  -.0002541   2.008881    -0.00   1.000    -3.937588     3.93708
-------------+----------------------------------------------------------------
_y_C         |
     des_CDN |   .2435073   2.376446     0.10   0.918     -4.41424    4.901255
      des_CN |  -.9279633   1.749824    -0.53   0.596    -4.357555    2.501629
       _cons |   1.177768   1.645018     0.72   0.474    -2.046408    4.401943
-------------+----------------------------------------------------------------
_y_D         |
     des_BDF |  -.5334962   2.268272    -0.24   0.814    -4.979228    3.912236
     des_CDN |   .8880329   2.843994     0.31   0.755    -4.686093    6.462159
      des_DH |  -1.768692   3.450895    -0.51   0.608    -8.532321    4.994937
    des_DHJN |   .2575521   2.530391     0.10   0.919    -4.701923    5.217028
      des_DN |  -.2604763    3.06597    -0.08   0.932    -6.269667    5.748714
       _cons |    .533242   2.268676     0.24   0.814    -3.913282    4.979766
-------------+----------------------------------------------------------------
_y_E         |
     des_EHN |  -.8455432   2.880088    -0.29   0.769    -6.490411    4.799325
      des_EL |    .000286    3.67666     0.00   1.000    -7.205836    7.206408
      des_EN |   .4981807   2.567788     0.19   0.846    -4.534591    5.530952
       _cons |  -.0002899   2.326651    -0.00   1.000    -4.560443    4.559863
-------------+----------------------------------------------------------------
_y_F         |
       _cons |  -.0002541    2.84051    -0.00   1.000    -5.567551    5.567042
-------------+----------------------------------------------------------------
_y_G         |
     des_AGN |   .0264071   1.742475     0.02   0.988    -3.388782    3.441596
     des_BGN |    1.31087   2.731332     0.48   0.631    -4.042442    6.664183
     des_GHN |   .2485301    1.75283     0.14   0.887    -3.186953    3.684013
     des_GKN |   .2991767   2.499671     0.12   0.905    -4.600089    5.198442
      des_GN |   -.242892   1.664428    -0.15   0.884    -3.505111    3.019327
       _cons |  -.0264071   1.422706    -0.02   0.985    -2.814859    2.762045
-------------+----------------------------------------------------------------
_y_H         |
    des_AHKN |   .4626025   2.846708     0.16   0.871    -5.116843    6.042048
     des_AHN |  -.4291491   2.277001    -0.19   0.851    -4.891989    4.033691
     des_BHI |  -1.004303   3.283197    -0.31   0.760     -7.43925    5.430644
     des_BHN |   .0609407   3.182669     0.02   0.985    -6.176976    6.298858
      des_CH |    .689633   2.877725     0.24   0.811    -4.950605    6.329871
    des_CHKL |    1.28896   3.281976     0.39   0.695    -5.143594    7.721514
    des_DHJN |  -.7217763   2.626757    -0.27   0.783    -5.870125    4.426573
     des_EHN |  -.7346285   2.631647    -0.28   0.780    -5.892562    4.423305
      des_GH |   .0847981    3.18077     0.03   0.979    -6.149396    6.318992
     des_GHN |   .0478484   2.222751     0.02   0.983    -4.308663     4.40436
      des_HN |  -.3677689   2.134914    -0.17   0.863    -4.552123    3.816585
       _cons |  -.1112046   2.010962    -0.06   0.956    -4.052617    3.830208
-------------+----------------------------------------------------------------
_y_I         |
       _cons |  -1.115449   2.595181    -0.43   0.667     -6.20191    3.971013
-------------+----------------------------------------------------------------
_y_J         |
      des_HJ |   .7146179   2.874053     0.25   0.804    -4.918422    6.347657
      des_IJ |  -2.506816   3.311201    -0.76   0.449     -8.99665    3.983019
      des_JL |  -.1615545    3.10832    -0.05   0.959     -6.25375    5.930641
      des_JN |   1.119759   2.008845     0.56   0.577    -2.817504    5.057022
       _cons |   .2727743   1.233938     0.22   0.825    -2.145699    2.691248
-------------+----------------------------------------------------------------
_y_K         |
      des_BK |  -1.893264   3.286745    -0.58   0.565    -8.335165    4.548637
    des_CHKL |   .3730593   3.287235     0.11   0.910    -6.069804    6.815922
      des_CK |   .4203662    3.28799     0.13   0.898    -6.023976    6.864709
     des_GKN |  -.5107617   2.881494    -0.18   0.859    -6.158387    5.136863
       _cons |   .7835312    2.01959     0.39   0.698    -3.174793    4.741856
-------------+----------------------------------------------------------------
_y_L         |
    des_CHKL |   1.156594   3.285162     0.35   0.725    -5.282205    7.595394
      des_LN |   1.371346   2.637422     0.52   0.603    -3.797906    6.540598
       _cons |  -3.92e-06   2.016214    -0.00   1.000    -3.951711    3.951703
-------------+----------------------------------------------------------------
_y_M         |
       _cons |   1.044049    2.39089     0.44   0.662    -3.642008    5.730107
-------------+----------------------------------------------------------------
_y_N         |
     des_ACN |  -.2340065   2.052718    -0.11   0.909    -4.257259    3.789246
     des_AGN |  -.2727762   1.089526    -0.25   0.802    -2.408209    1.862656
    des_AHKN |   .5663249   2.063195     0.27   0.784    -3.477463    4.610113
     des_AHN |  -.7919424   1.147179    -0.69   0.490    -3.040372    1.456487
      des_AN |  -.3243747   .5577011    -0.58   0.561    -1.417449    .7686993
    des_BDEN |  -1.393902   2.630796    -0.53   0.596    -6.550166    3.762363
     des_BGN |    .838804   2.380892     0.35   0.725    -3.827659    5.505267
     des_BHN |  -.2983475   2.502191    -0.12   0.905    -5.202552    4.605857
      des_BN |   .0261755   2.108412     0.01   0.990    -4.106237    4.158588
       _cons |   .2727762   .4182577     0.65   0.514    -.5469937    1.092546
------------------------------------------------------------------------------

Estimated between-studies SDs and correlation matrix:
           SD        _y_B        _y_C        _y_D        _y_E        _y_F        _y_G        _y_H        _y_I        _y_J        _y_K
_y_B  1.149e-09           1           .           .           .           .           .           .           .           .           .
_y_C  1.149e-09          .5           1           .           .           .           .           .           .           .           .
_y_D  1.149e-09          .5          .5           1           .           .           .           .           .           .           .
_y_E  1.149e-09          .5          .5          .5           1           .           .           .           .           .           .
_y_F  1.149e-09          .5          .5          .5          .5           1           .           .           .           .           .
_y_G  1.149e-09          .5          .5          .5          .5          .5           1           .           .           .           .
_y_H  1.149e-09          .5          .5          .5          .5          .5          .5           1           .           .           .
_y_I  1.149e-09          .5          .5          .5          .5          .5          .5          .5           1           .           .
_y_J  1.149e-09          .5          .5          .5          .5          .5          .5          .5          .5           1           .
_y_K  1.149e-09          .5          .5          .5          .5          .5          .5          .5          .5          .5           1
_y_L  1.149e-09          .5          .5          .5          .5          .5          .5          .5          .5          .5          .5
_y_M  1.149e-09          .5          .5          .5          .5          .5          .5          .5          .5          .5          .5
_y_N  1.149e-09          .5          .5          .5          .5          .5          .5          .5          .5          .5          .5

            _y_L        _y_M        _y_N
_y_B           .           .           .
_y_C           .           .           .
_y_D           .           .           .
_y_E           .           .           .
_y_F           .           .           .
_y_G           .           .           .
_y_H           .           .           .
_y_I           .           .           .
_y_J           .           .           .
_y_K           .           .           .
_y_L           1           .           .
_y_M          .5           1           .
_y_N          .5          .5           1

Testing for inconsistency:
 ( 1)  [_y_B]des_ABN = 0
 ( 2)  [_y_N]des_ACN = 0
 ( 3)  [_y_N]des_AGN = 0
 ( 4)  [_y_G]des_AGN = 0
 ( 5)  [_y_N]des_AHKN = 0
 ( 6)  [_y_H]des_AHKN = 0
 ( 7)  [_y_N]des_AHN = 0
 ( 8)  [_y_H]des_AHN = 0
 ( 9)  [_y_N]des_AN = 0
 (10)  [_y_N]des_BDEN = 0
 (11)  [_y_D]des_BDF = 0
 (12)  [_y_N]des_BGN = 0
 (13)  [_y_G]des_BGN = 0
 (14)  [_y_H]des_BHI = 0
 (15)  [_y_N]des_BHN = 0
 (16)  [_y_H]des_BHN = 0
 (17)  [_y_K]des_BK = 0
 (18)  [_y_N]des_BN = 0
 (19)  [_y_C]des_CDN = 0
 (20)  [_y_D]des_CDN = 0
 (21)  [_y_H]des_CH = 0
 (22)  [_y_H]des_CHKL = 0
 (23)  [_y_K]des_CHKL = 0
 (24)  [_y_L]des_CHKL = 0
 (25)  [_y_K]des_CK = 0
 (26)  [_y_C]des_CN = 0
 (27)  [_y_D]des_DH = 0
 (28)  [_y_H]des_DHJN = 0
 (29)  [_y_D]des_DHJN = 0
 (30)  [_y_D]des_DN = 0
 (31)  [_y_H]des_EHN = 0
 (32)  [_y_E]des_EHN = 0
 (33)  [_y_E]des_EL = 0
 (34)  [_y_E]des_EN = 0
 (35)  [_y_H]des_GH = 0
 (36)  [_y_G]des_GHN = 0
 (37)  [_y_H]des_GHN = 0
 (38)  [_y_G]des_GKN = 0
 (39)  [_y_K]des_GKN = 0
 (40)  [_y_G]des_GN = 0
 (41)  [_y_J]des_HJ = 0
 (42)  [_y_H]des_HN = 0
 (43)  [_y_J]des_IJ = 0
 (44)  [_y_J]des_JL = 0
 (45)  [_y_J]des_JN = 0
 (46)  [_y_L]des_LN = 0

           chi2( 46) =    9.64
         Prob > chi2 =    1.0000
```

Supplement Figure 8. Inconsistency model for DVT rate
