# Supplementary material for: Effects of Tranexamic Acid on Hemorrhage Control and Deep Venous Thrombosis Rate After Total Knee Arthroplasty: A Systematic Review and Network Meta-Analysis of Randomized Controlled Trials
Source: Front Pharmacol. 2021 Jul 21;12:639694. doi: 10.3389/fphar.2021.639694 (PMC8335562; doi:10.3389/fphar.2021.639694)
Supplement: Supplementary file 8 [file Image4.pdf]

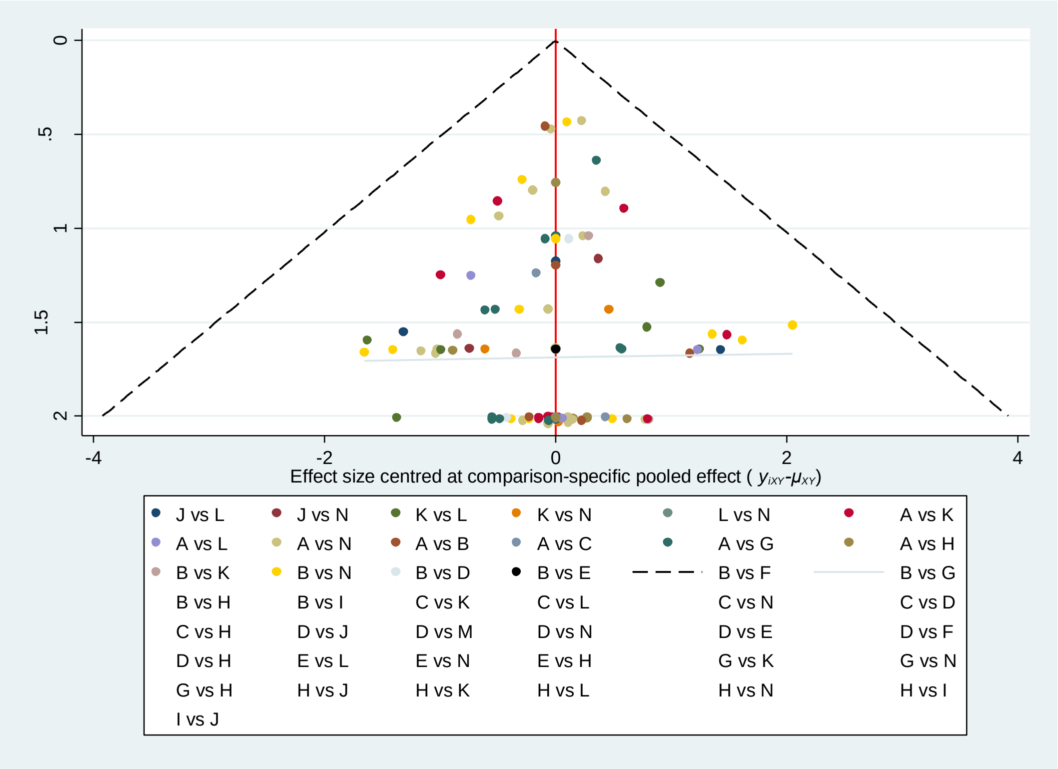

Supplement Figure 4. The inverted funnel plot for DVT rate. (A: IV TXA ≤ 10mg/kg or 1g once; B: IV TXA ≥ 15mg/kg or 1g once; C: IV TXA ≤ 10mg/kg or 1g twice; D: IV TXA ≥ 15mg/kg or 1g twice; E: IV TXA ≤ 10mg/kg or 1g three times; F: IV TXA ≥ 15mg/kg or 1g three times; G: IA TXA < 2g; H: IA TXA ≥ 2g; I: oral TXA ≤ 2g; J: oral TXA > 2g; K: IV/IV infusion + IA TXA ≤ 3g; L: IV/IV infusion + IA TXA > 3g; M: IV/IV infusion + oral TXA > 3g)
