# Supplementary material for: Effects of Tranexamic Acid on Hemorrhage Control and Deep Venous Thrombosis Rate After Total Knee Arthroplasty: A Systematic Review and Network Meta-Analysis of Randomized Controlled Trials
Source: Front Pharmacol. 2021 Jul 21;12:639694. doi: 10.3389/fphar.2021.639694 (PMC8335562; doi:10.3389/fphar.2021.639694)
Supplement: Supplementary file 9 [file Image2.pdf]

	Random sequence generation (selection bias)	Allocation concealment (selection bias)	Blinding of participants and personnel (performance bias)	Blinding of outcome assessment (detection bias)	Incomplete outcome data (attrition bias)	Selective reporting (reporting bias)	Other bias
Adravanti2018	+	+	+	+	+	+	+
Aguilera2013	+	+	+	+	+	+	+
Aguilera2015	+	+	+	+	+	+	+
Almeida2018	+	+	+	+	+	+	+
Alshryda2013	+	+	+	+	+	+	+
Antinolf2014	+	+	+	+	+	+	+
Benoni1997	+	?	+	+	+	−	+
Bidolegui2014	+	+	+	+	+	+	+
Camarasa2006	+	+	+	+	+	+	+
Carvalho2015	+	−	−	+	+	+	+
Castro-Menendez2016	?	−	−	−	+	+	−
Chen2016	+	+	+	+	+	+	+
Drosos2016	+	−	−	+	+	+	+
Fillingham2016	+	+	+	+	+	+	+
George2018	+	+	+	+	+	+	+
Georgiadis2013	+	+	+	+	+	+	+
Gomez-Barrena2014	+	+	+	+	+	+	+
Good2003	+	+	+	+	+	+	+
Guzel2016	+	−	−	+	+	+	+
Hiippala1995	+	+	+	+	+	+	+
Huang2014	+	+	+	+	+	+	+
Huang2017	+	+	+	+	+	+	+
Kakar2009	+	+	+	+	+	+	+
Karaaslan2015	+	+	+	+	+	+	+
Keyhani2016	+	−	−	+	+	+	?
Kim2014	+	+	+	+	+	+	+
King2019	+	+	+	+	+	+	+
Kundu2015	+	+	+	+	+	+	+
Kwok2018	−	−	−	+	+	+	+
Lee2013	+	+	+	+	+	+	+
Lee2017a	+	+	+	+	+	+	+
Lee2017b	+	?	+	+	+	+	+
Levine2014	+	+	+	+	+	+	+
Lin2012	+	+	+	+	+	+	+
Lin2015	+	−	−	+	+	+	+
Liu2018	?	?	+	+	+	+	+
MacGillivray2011	+	+	+	+	+	+	+
Maniar2012	+	+	+	+	+	+	+
Martin2014	+	+	+	+	+	+	+
McConnell2012	+	+	+	+	+	+	+
Mehta2018	+	−	−	+	+	+	+
Molloy2007	+	+	+	+	+	+	+
Motififard2015	+	+	+	+	+	+	+
Nambiar2019	+	+	+	+	+	+	+
Nielsen2016	+	+	+	+	+	+	+
Orpen2006	+	+	+	+	+	+	+
Oztaş2015	+	?	−	+	+	+	+
Patel2014	+	−	−	+	+	+	+
Prakash2017	+	+	+	+	+	+	+
Sangasoongsong2011	+	+	+	+	+	+	+
Sangasoongsong2013	+	+	+	+	+	+	+
Sarzaeem2014	+	?	+	+	+	+	+
Seo2013	+	+	+	+	+	+	+
Seviciu2016	+	+	+	+	+	+	+
Shen2015	+	+	+	+	+	+	+
Shinde2015	+	+	+	+	+	+	+
Song2017	+	+	+	+	+	+	+
Stowers2017	+	+	+	+	+	+	+
Sun2017	+	−	−	+	+	+	+
Takuya2018	+	+	+	+	+	+	+
Tammachote2019	+	+	+	+	+	+	+
Tanaka2001	?	+	+	+	+	+	+
Tang2019	+	+	+	+	+	+	+
Tzatzairis2016	+	−	−	+	+	+	?
Tzatzairis2019	+	+	+	+	+	+	+
Ugurlu2017	+	−	−	+	+	+	+
Veien2002	+	+	+	+	+	+	+
Volquind2016	+	+	+	+	+	+	+
Wang2015a	+	−	−	+	+	+	+
Wang2015b	+	+	+	+	+	+	+
Wang2017a	+	+	+	+	+	+	+
Wang2017b	+	+	+	+	+	+	+
Wang2018a	+	+	+	+	+	+	+
Wang2018b	+	+	+	+	+	+	+
Wang2019	+	+	+	+	+	+	+
Wong2010	+	+	+	+	+	+	+
Xu2016	+	+	+	+	+	+	+
Yang2015	+	?	+	+	+	+	+
Yen2017	+	+	+	+	+	+	?
Yuan2017	+	?	+	+	+	+	+
Zekcer2016	+	−	−	+	+	+	+

Supplement Figure 2. Risk of Bias Summary
