# Supplementary material for: Effects of Tranexamic Acid on Hemorrhage Control and Deep Venous Thrombosis Rate After Total Knee Arthroplasty: A Systematic Review and Network Meta-Analysis of Randomized Controlled Trials
Source: Front Pharmacol. 2021 Jul 21;12:639694. doi: 10.3389/fphar.2021.639694 (PMC8335562; doi:10.3389/fphar.2021.639694)
Supplement: Supplementary file 11 [file Table2.docx]

Supplement Table 2. Meta-regression results

| Covariate | mean | SD | 95%CI |
| --- | --- | --- | --- |
| Age | 100.18 | 50.85 | (1.70, 201.07) |
| BMI | -37.31 | 62.17 | (-159.12, 84.88) |
| Unilateral | -38.02 | 97.38 | (-228.50, 153.015) |
| Tourniquet | 48.47 | 85.26 | (-120.9, 214.0) |

BMI: Body Mass Index; SD, standard deviation; CI, Confidence Interval
