# Supplementary material for: Effects of Tranexamic Acid on Hemorrhage Control and Deep Venous Thrombosis Rate After Total Knee Arthroplasty: A Systematic Review and Network Meta-Analysis of Randomized Controlled Trials
Source: Front Pharmacol. 2021 Jul 21;12:639694. doi: 10.3389/fphar.2021.639694 (PMC8335562; doi:10.3389/fphar.2021.639694)
Supplement: Supplementary file 12 [file Image7.pdf]

```
. network meta i
Command is: mvmeta _y _S  , bscovariance(exch 0.5) longparm suppress(uv mm) eq(_y_B: des_BDEN des_BGN des_BHI des_BK des_BN, _y_C: d
> es_ACN, _y_D: des_BDF des_DH, _y_G: des_AGN des_BGN des_EGLN des_GHN des_GKN des_GN, _y_H: des_AHN des_CHKL des_GH des_GHN des_HN,
>  _y_I: des_BHI, _y_J: des_IJ des_JL des_JN, _y_K: des_CHKL des_CK des_GKN, _y_L: des_CHKL des_EGLN des_EL des_LN, _y_N: des_ACN de
> s_AGN des_AHKN des_AHN des_AN des_BCN des_BDEN des_CN des_EGLN des_EN) vars(_y_B _y_C _y_D _y_E _y_F _y_G _y_H _y_I _y_J _y_K _y_L
>  _y_M _y_N)
Note: using method reml
Note: regressing _y_B on des_BDEN des_BGN des_BHI des_BK des_BN
Note: regressing _y_C on des_ACN
Note: regressing _y_D on des_BDF des_DH
Note: regressing _y_E on (nothing)
Note: regressing _y_F on (nothing)
Note: regressing _y_G on des_AGN des_BGN des_EGLN des_GHN des_GKN des_GN
Note: regressing _y_H on des_AHN des_CHKL des_GH des_GHN des_HN
Note: regressing _y_I on des_BHI
Note: regressing _y_J on des_IJ des_JL des_JN
Note: regressing _y_K on des_CHKL des_CK des_GKN
Note: regressing _y_L on des_CHKL des_EGLN des_EL des_LN
Note: regressing _y_M on (nothing)
Note: regressing _y_N on des_ACN des_AGN des_AHKN des_AHN des_AN des_BCN des_BDEN des_CN des_EGLN des_EN
Note: 65 observations on 13 variables
Note: variance-covariance matrix is proportional to .5*I(13)+.5*J(13,13,1)

initial:       log likelihood = -927.20453
rescale:       log likelihood = -927.20453
rescale eq:    log likelihood = -688.07181
Iteration 0:   log likelihood = -688.07181
Iteration 1:   log likelihood = -683.79202
Iteration 2:   log likelihood = -683.67627
Iteration 3:   log likelihood = -683.67585
Iteration 4:   log likelihood = -683.67585

Multivariate meta-analysis
Variance-covariance matrix = proportional .5*I(13)+.5*J(13,13,1)
Method = reml                                   Number of dimensions    =    13
Restricted log likelihood = -683.67585          Number of observations  =    65
------------------------------------------------------------------------------
             |      Coef.   Std. Err.      z    P>|z|     [95% Conf. Interval]
-------------+----------------------------------------------------------------
_y_B         |
    des_BDEN |  -29.43255   335.1634    -0.09   0.930    -686.3407    627.4756
     des_BGN |  -163.8479   324.4064    -0.51   0.614    -799.6729     471.977
     des_BHI |  -132.7886   386.5071    -0.34   0.731    -890.3287    624.7515
      des_BK |  -141.7966   382.3309    -0.37   0.711    -891.1515    607.5583
      des_BN |  -129.1314   307.4376    -0.42   0.674    -731.6981    473.4353
       _cons |   109.9566    279.889     0.39   0.694    -438.6158     658.529
-------------+----------------------------------------------------------------
_y_C         |
     des_ACN |  -87.13761   265.8084    -0.33   0.743    -608.1125    433.8373
       _cons |   38.13761    188.046     0.20   0.839    -330.4258     406.701
-------------+----------------------------------------------------------------
_y_D         |
     des_BDF |   125.2687   382.0155     0.33   0.743     -623.468    874.0054
      des_DH |   95.02585   384.1915     0.25   0.805    -657.9757    848.0274
       _cons |  -115.3024   260.0502    -0.44   0.657    -624.9915    394.3866
-------------+----------------------------------------------------------------
_y_E         |
       _cons |  -137.5812   187.7759    -0.73   0.464    -505.6152    230.4528
-------------+----------------------------------------------------------------
_y_F         |
       _cons |  -200.0337   334.6933    -0.60   0.550    -856.0205     455.953
-------------+----------------------------------------------------------------
_y_G         |
     des_AGN |  -41.28009   210.4265    -0.20   0.844    -453.7084    371.1482
     des_BGN |  -72.64433   303.1872    -0.24   0.811    -666.8804    521.5917
    des_EGLN |  -67.44302   320.1941    -0.21   0.833     -695.012     560.126
     des_GHN |   26.66491   302.0789     0.09   0.930    -565.3989    618.7287
     des_GKN |   70.08602   332.0445     0.21   0.833    -580.7093    720.8813
      des_GN |  -12.45189   295.0145    -0.04   0.966    -590.6698     565.766
       _cons |  -44.27446   179.7989    -0.25   0.805    -396.6738    308.1249
-------------+----------------------------------------------------------------
_y_H         |
     des_AHN |   40.35271   210.8851     0.19   0.848    -372.9745    453.6799
    des_CHKL |  -21.05751   319.0711    -0.07   0.947    -646.4254    604.3104
      des_GH |  -342.4344   318.3982    -1.08   0.282    -966.4834    281.6146
     des_GHN |   5.816821   303.7409     0.02   0.985    -589.5044    601.1381
      des_HN |  -56.95127   308.4224    -0.18   0.854     -661.448    547.5455
       _cons |  -71.78045   182.5171    -0.39   0.694    -429.5074    285.9465
-------------+----------------------------------------------------------------
_y_I         |
     des_BHI |   -177.174   330.1117    -0.54   0.591     -824.181     469.833
       _cons |   50.02195   193.9682     0.26   0.796    -330.1488    430.1927
-------------+----------------------------------------------------------------
_y_J         |
      des_IJ |  -30.30195   379.1196    -0.08   0.936    -773.3628    712.7589
      des_JL |  -98.85164   395.1656    -0.25   0.802     -873.362    675.6587
      des_JN |   196.8447   381.0706     0.52   0.605    -550.0399    943.7293
       _cons |  -155.3654   263.1874    -0.59   0.555    -671.2032    360.4723
-------------+----------------------------------------------------------------
_y_K         |
    des_CHKL |   38.01452   319.2261     0.12   0.905    -587.6572    663.6863
      des_CK |   39.99361   321.7919     0.12   0.901     -590.707    670.6942
     des_GKN |   21.26407   333.9953     0.06   0.949    -633.3548    675.8829
       _cons |  -121.8525   182.3372    -0.67   0.504    -479.2268    235.5218
-------------+----------------------------------------------------------------
_y_L         |
    des_CHKL |    211.035   338.6524     0.62   0.533    -452.7116    874.7816
    des_EGLN |   209.1655   339.7516     0.62   0.538    -456.7354    875.0664
      des_EL |   127.6315   341.9087     0.37   0.709    -542.4971    797.7602
      des_LN |  -207.3707   354.7111    -0.58   0.559    -902.5917    487.8504
       _cons |  -372.8729    214.539    -1.74   0.082    -793.3617    47.61579
-------------+----------------------------------------------------------------
_y_M         |
       _cons |  -359.3566   320.7713    -1.12   0.263    -988.0569    269.3437
-------------+----------------------------------------------------------------
_y_N         |
     des_ACN |  -82.53084   278.4351    -0.30   0.767    -628.2536    463.1919
     des_AGN |  -51.39214   234.9004    -0.22   0.827    -511.7885    409.0042
    des_AHKN |    104.435   288.8455     0.36   0.718    -461.6917    670.5617
     des_AHN |  -18.11694   232.4799    -0.08   0.938    -473.7692    437.5353
      des_AN |   104.2523    215.248     0.48   0.628     -317.626    526.1306
     des_BCN |   231.7635   301.5818     0.77   0.442    -359.3259    822.8529
    des_BDEN |   230.6232    279.828     0.82   0.410    -317.8297     779.076
      des_CN |   160.4313   223.6988     0.72   0.473    -278.0103    598.8729
    des_EGLN |  -258.2483   282.3339    -0.91   0.360    -811.6125    295.1159
      des_EN |    188.991   266.2383     0.71   0.478    -332.8265    710.8085
       _cons |   269.5308   206.5697     1.30   0.192    -135.3384    674.4001
------------------------------------------------------------------------------

Estimated between-studies SDs and correlation matrix:
            SD        _y_B        _y_C        _y_D        _y_E        _y_F        _y_G        _y_H        _y_I        _y_J        _y_K
_y_B  179.45083          1           .           .           .           .           .           .           .           .           .
_y_C  179.45083         .5           1           .           .           .           .           .           .           .           .
_y_D  179.45083         .5          .5           1           .           .           .           .           .           .           .
_y_E  179.45083         .5          .5          .5           1           .           .           .           .           .           .
_y_F  179.45083         .5          .5          .5          .5           1           .           .           .           .           .
_y_G  179.45083         .5          .5          .5          .5          .5           1           .           .           .           .
_y_H  179.45083         .5          .5          .5          .5          .5          .5           1           .           .           .
_y_I  179.45083         .5          .5          .5          .5          .5          .5          .5           1           .           .
_y_J  179.45083         .5          .5          .5          .5          .5          .5          .5          .5           1           .
_y_K  179.45083         .5          .5          .5          .5          .5          .5          .5          .5          .5           1
_y_L  179.45083         .5          .5          .5          .5          .5          .5          .5          .5          .5          .5
_y_M  179.45083         .5          .5          .5          .5          .5          .5          .5          .5          .5          .5
_y_N  179.45083         .5          .5          .5          .5          .5          .5          .5          .5          .5          .5

            _y_L        _y_M        _y_N
_y_B           .           .           .
_y_C           .           .           .
_y_D           .           .           .
_y_E           .           .           .
_y_F           .           .           .
_y_G           .           .           .
_y_H           .           .           .
_y_I           .           .           .
_y_J           .           .           .
_y_K           .           .           .
_y_L           1           .           .
_y_M          .5           1           .
_y_N          .5          .5           1

Testing for inconsistency:
 ( 1)  [_y_C]des_ACN = 0
 ( 2)  [_y_N]des_ACN = 0
 ( 3)  [_y_N]des_AGN = 0
 ( 4)  [_y_G]des_AGN = 0
 ( 5)  [_y_N]des_AHKN = 0
 ( 6)  [_y_N]des_AHN = 0
 ( 7)  [_y_H]des_AHN = 0
 ( 8)  [_y_N]des_AN = 0
 ( 9)  [_y_N]des_BCN = 0
 (10)  [_y_N]des_BDEN = 0
 (11)  [_y_B]des_BDEN = 0
 (12)  [_y_D]des_BDF = 0
 (13)  [_y_G]des_BGN = 0
 (14)  [_y_B]des_BGN = 0
 (15)  [_y_I]des_BHI = 0
 (16)  [_y_B]des_BHI = 0
 (17)  [_y_B]des_BK = 0
 (18)  [_y_B]des_BN = 0
 (19)  [_y_H]des_CHKL = 0
 (20)  [_y_K]des_CHKL = 0
 (21)  [_y_L]des_CHKL = 0
 (22)  [_y_K]des_CK = 0
 (23)  [_y_N]des_CN = 0
 (24)  [_y_D]des_DH = 0
 (25)  [_y_N]des_EGLN = 0
 (26)  [_y_G]des_EGLN = 0
 (27)  [_y_L]des_EGLN = 0
 (28)  [_y_L]des_EL = 0
 (29)  [_y_N]des_EN = 0
 (30)  [_y_H]des_GH = 0
 (31)  [_y_G]des_GHN = 0
 (32)  [_y_H]des_GHN = 0
 (33)  [_y_G]des_GKN = 0
 (34)  [_y_K]des_GKN = 0
 (35)  [_y_G]des_GN = 0
 (36)  [_y_H]des_HN = 0
 (37)  [_y_J]des_IJ = 0
 (38)  [_y_J]des_JL = 0
 (39)  [_y_J]des_JN = 0
 (40)  [_y_L]des_LN = 0

           chi2( 40) =   20.09
         Prob > chi2 =    0.9964
mvmeta command stored as F9; test command stored as F8
```

Supplement Figure 7. Inconsistency model for total blood loss
