# Supplementary figures and images for: Effects of Tranexamic Acid on Hemorrhage Control and Deep Venous Thrombosis Rate After Total Knee Arthroplasty: A Systematic Review and Network Meta-Analysis of Randomized Controlled Trials
Source: Front Pharmacol. 2021 Jul 21;12:639694. doi: 10.3389/fphar.2021.639694 (PMC8335562; doi:10.3389/fphar.2021.639694)

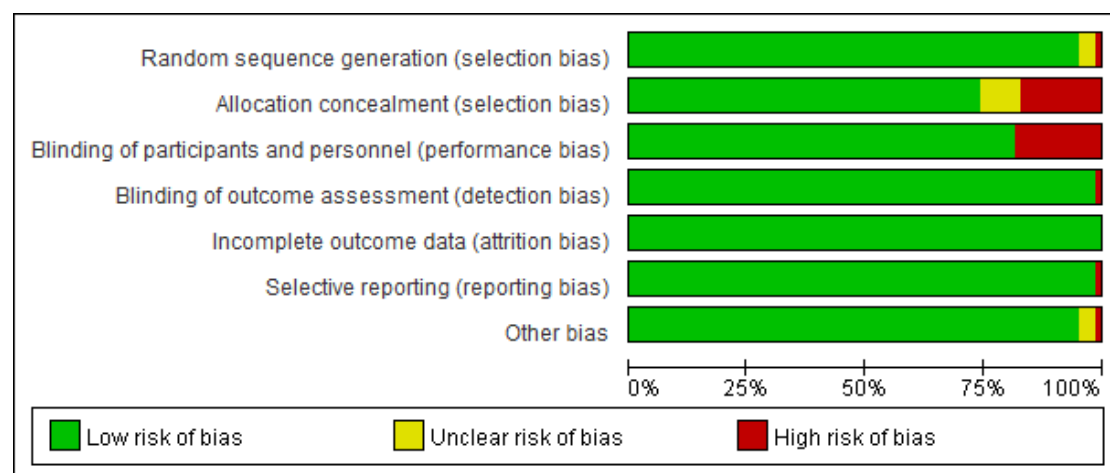

Supplement Figure 1. Risk of Bias Graph.

Supplement: Supplementary file 13 [file Image1.pdf]
